# Brassinosteroids Affect the Symbiosis Between the AM Fungus *Rhizoglomus irregularis* and Solanaceous Host Plants

**DOI:** 10.3389/fpls.2019.00571

**Published:** 2019-05-15

**Authors:** Lea von Sivers, Hannah Jaspar, Bettina Johst, Michael Roese, Michael Bitterlich, Philipp Franken, Christina Kühn

**Affiliations:** ^1^Plant Physiology, Institute of Biology, Humboldt University of Berlin, Berlin, Germany; ^2^Leibniz-Institute of Vegetable and Ornamental Crops, Großbeeren, Germany; ^3^Erfurt Research Centre for Horticultural Crops, University of Applied Sciences Erfurt, Erfurt, Germany

**Keywords:** BR signaling, sucrose transport, arbuscular mycorrhiza, endocytosis, SUT2, MSBP1, *Solanum lycopersicum*, *Nicotiana tabacum*

## Abstract

Together with several proteins involved in brassinosteroid (BR) signaling and synthesis, the membrane steroid binding protein 1 (MSBP1) was identified within the interactome of the sucrose transporter of tomato (SlSUT2). We asked whether MSBP1 is also involved in BR signaling as assumed for the AtMSBP1 protein from Arabidopsis and whether it impacts root colonization with arbuscular mycorrhizal (AM) fungi in a similar way as shown previously for SlSUT2. In addition, we asked whether brassinosteroids *per se* affect efficiency of root colonization by AM fungi. We carried out a set of experiments with transgenic tobacco plants with increased and decreased MSBP1 expression levels. We investigated the plant and the mycorrhizal phenotype of these transgenic plants and tested the involvement of MSBP1 in BR metabolism by application of epi-brassinolide and brassinazole, an inhibitor of BR biosynthesis. We show that the phenotype of the transgenic tobacco plants with increased or reduced *MSBP1* expression is consistent with an inhibitory role of MSBP1 in BR signaling. *MSBP1* overexpression could be mimicked by brassinazole treatment. Interestingly, manipulation of *MSBP1* expression in transgenic tobacco plants not only affected plant growth and development, but also the host plant responses toward colonization with AM fungi, as well as arbuscular architecture. Moreover, we observed that brassinosteroids indeed have a direct impact on the nutrient exchange in AM symbiosis and on the biomass production of colonized host plants. Furthermore, arbuscular morphology is affected by changes in *MSBP1* expression and brassinolide or brassinazole treatments. We conclude that host plant growth responses and nutrient exchange within the symbiosis with AM fungi is controlled by brassinosteroids and might be impeded by the MSBP1 protein.

**Highlights**

- Behavior of transgenic tobacco plants with up- or down-regulated *MSBP1* expression is consistent with an inhibitory role of MSBP1 in BR signaling.
- We show for the first time that brassinosteroids *per se* positively affect the symbiosis of tobacco and tomato plants with an arbuscular mycorrhizal fungus.
- MSBP1 expression, and brassinolide treatment affects arbuscular morphology as well as host plant growth responses.

## Introduction

Brassinosteroids are phytohormones involved in cell elongation and expansion, in pollen and stamen development (Clouse and Sasse, 1998; Zhu et al., 2015), as well as in fruit ripening (Symons et al., 2006). The physiological role of brassinosteroids (BRs) in plant development, formation of the vascular system and pollen maturation has been investigated intensively during the past few years. In addition, a role in plant defense against pathogens could be shown (Zhang et al., 2015). However, it is still unclear whether the inhibitory role of BRs in defense responses against plant pathogens is also effective in other biotic interactions such as mycorrhizal symbioses. Epi-brassinolide treatment in wheat and rice has slight synergistic effects on mycorrhization under salt stress conditions (Tofighi et al., 2017). Tomato *dx* mutants defective in BR biosynthesis showed decreased mycorrhization (Bitterlich et al., 2014a,b). BR-deficient *lkb* pea mutants, however, did not show changes in root colonization (Foo et al., 2013) suggesting species-dependent differences.

We recently showed that the tomato sucrose transporter SlSUT2 directly interacts with membrane proteins involved in BR synthesis and signaling. These interaction partners were the sterol reductase DWARF1/DIMINUTO (DIM1) involved in BR synthesis, the BR co-receptor kinase BAK1, and the less well characterized membrane-steroid binding protein 1 (MSBP1) (Bitterlich et al., 2014b). Interaction between BAK1 and MSBP1 has been already described in Arabidopsis (Song et al., 2009) and bimolecular fluorescence complementation (BiFC) as well as the yeast two-hybrid split ubiquitin system (SUS) were able to confirm this interaction also for the two tomato paralogs interacting with the sucrose transporter SlSUT2 (Bitterlich et al., 2014b). Whether all three proteins are able to form a trimeric complex or whether they compete with each other for binding, is unknown. It has been suggested that the interaction between BAK1 and MSBP1 inhibits activation of the BR receptor BRI1 by BAK1 (Song et al., 2009).

MSBP1 and the BAK1 protein were identified as the two strongest interaction partners of SlSUT2. Inhibition of *SlSUT2* expression in transgenic tomato plants leads to delayed fruit development, reduced pollen germination rates and inhibited pollen tube growth (Hackel et al., 2006), whereas mycorrhizal colonization of *SlSUT2*-antisense plants is significantly increased (Bitterlich et al., 2014b). Apart from mycorrhization intensity, the phenotype of *SlSUT2*-inhibited plants resembles BR deficient mutant plants.

BAK1 is well characterized and the promiscuous leucine rich repeat (LRR) receptor kinase interacts with BRI1 in BR signaling and with other receptor kinases in plant immune responses and cell death pathway (Belkhadir et al., 2012; Segonzac et al., 2014).

The role of MSBP1 is, however, only partially understood. In Arabidopsis, MSBP1 binds to progesterone, stigmasterol and brassinosteroid with low affinity (Yang et al., 2005) and is thought to inhibit BR signaling by removing BAK1 from the plasma membrane by internalization as heteromeric complexes (Song et al., 2009). In Arabidopsis, MSBP1 is light induced (Shi et al., 2011) and involved in vesicle trafficking and auxin redistribution (Yang et al., 2008). MSBP1 inhibits cell elongation (Yang et al., 2005), potentially via its inhibitory effect on BR signaling. A recent publication describes MSBP1 to be important for salinity tolerance in yeast cells, and in Arabidopsis and barley (Witzel et al., 2018). In barley, MSBP1 abundance correlates with adaptation of root architecture in response to salinity, potentially via stimulation of auxin response through PIN2 homologs and enhancement of BR receptor endocytosis, thereby reducing BR signaling (Witzel et al., 2018). A critical role of MSBP1 is postulated for mycorrhiza development in Medicago roots after its induction by a diffusible signal emitted by the fungus (Kuhn et al., 2010). Thus, the MSBP1 protein has multiple functions and is still not fully understood in detail.

To further elucidate the impact of MSBP1 on plant development, its relation to brassinosteroid signaling and its role in the mycorrhizal symbiosis, we analyzed the development of tobacco plants with increased or decreased *MSBP1*-expression. For overexpression, we used the homologous gene of tomato in order to avoid any down-regulation of RNA accumulation due to co-suppression. Secondly, we conducted a detailed study of the arbuscular mycorrhizal (AM) phenotype in these transgenic plants including an analysis of arbuscule morphology. In order to test the hypothesis that MSBP1 is involved in BR signaling, we studied the effects of treatments with the synthetic BR epi-brassinolide and the BR inhibitor brassinazole on the plant and AM phenotype.

## Materials and Methods

### Generation of Constructs

The open reading frame of the MSBP1-like protein from tomato (XP_004240738) comprises 723 bp and encodes a 240 amino acids protein (Supplementary Figure S1). A 127 bp *Xba*I-*Bgl*II fragment was used for cloning into the pUC-RNAi vector (Chen et al., 2003) in sense and antisense orientation. The whole 454 bp fragment including the intron sequence was excised with *Pst*I and transferred into the binary vector pBinAR Kan linearized with *Sbf*I. The construct was checked by sequencing. Sequence similarity between the tomato (XM_004240690) and the tobacco ortholog (XM_016639423) within the *XbaI-BglII* fragment is 91% identity.

The full length cDNA of MSBP1 (XP_004240738) was amplified via PCR from reversely transcribed RNA isolated from tomato leaves or flowers using primers carrying *Kpn*I- and *BamH*I restriction sites (Supplementary Table S1). Ligation into the binary vector was carried out via *Kpn*I-*BamH*I in sense orientation followed by sequence analysis.

### Transformation of *Nicotiana tabacum* via *Agrobacterium tumefaciens*

Gene transfer into plants (*Nicotiana tabacum* var. Samsun SNN) was performed with *Agrobacterium tumefaciens* (strain C58C1, pGV2260; Deblaere et al., 1985). Regenerated plants were screened by PCR for integration of the construct using NPTII primers (Supplementary Table S1). Seeds of plants containing integrated DNA were selected in tissue culture on selective media with the appropriate antibiotics and placed in the greenhouse for further analysis.

### Plant Growth

Tobacco and tomato (*Solanum lycopersicum* cv. Moneymaker) plants were grown in the greenhouse with a cycle of 16 h light (22°C) and 8 h darkness (15°C). The mean photosynthetic photon flux density was about 150 μmol photons m^-2^ s^-1^ and additional illumination was provided by high-pressure sodium lamps SON-T Green Power and metal halide lamps MASTER LPI-T Plus (Philips Belgium, Brussels).

### Epi-Brassinolide and Brassinazole Treatments

Epibrassinolide (epi-BL; ≤85%. Sigma-Aldrich) and brassinazole (BRZ; ≤98% (HPLC), Sigma-Aldrich) were dissolved in DMSO and freshly diluted in water to a final concentration of 1 μM (Asami et al., 2000). Both chemicals were applied to the root system of tobacco plants starting 1 week after inoculation. Fifty milliliter of 1 μM BRZ was applied every other day, whereas control (supplemented with the same amount of DMSO) and epi-BL was applied weekly over a total period of 4 weeks.

### Mycorrhization Experiments

For mycorrhization experiments, seedlings of tobacco and tomato wild type plants and the corresponding transgenic lines were planted in 3 L pots with a sand-vermiculite mixture (1:1; agra-Vermiculite, RHP, TX Rhenen, the Netherlands; quarry sand grain size: 0.2–1.0 mm, Euroquarz, Ottendorf-Okrilla, Germany). Half of the pots were inoculated with *Rhizoglomus irregularis* QS 81 (INOQ, Schnega, Germany) in a ratio of 1:10 inoculum:substrate. The control plants were grown in the same substrate without inoculum. The plants were grown in the greenhouse in a randomized design and watered every day with 200–400 ml of nutrient solution (De Kreji et al., 1997), containing 10% of the standard phosphate concentration (0.1 mM). Plants were harvested after 6 weeks if not indicated otherwise. Samples of roots and leaves were taken in liquid nitrogen and stored at –80°C. For determination of dry mass, plants were dried for 4 days at 65°C. Approximately 1 g of each fresh root system was stored in 20% EtOH for later analysis of fungal colonization.

The root samples were stained with trypan blue after (Koske and Gemma, 1989). Scoring of colonization patterns was determined after the method of Trouvelot et al. (1986) and calculations were carried out with the MYCOCALC software^1^. Quantification of fungal vesicles was carried out according to the classification for arbuscule abundance described in Trouvelot et al. (1986) Alternatively, roots were stained with wheat germ agglutinin (WGA). WGA-FITC (Sigma-Aldrich, St. Louis, MO, United States) staining was performed over night at a concentration of 10 μg ml^-1^ in PBS after clearing to the roots for 2 h at 50°C in 10% KOH as described by Banhara et al. (2015). Root samples were imaged with TCS-SP2 (Leica, Bensheim, Germany) or LSM800 (Zeiss, Jena, Germany) confocal microscopes with excitation at 488 nm for WGA-FITC and detection at 500–540 nm. Tubule diameter was quantified from 20 to 80 tubules per arbuscule and 10–15 arbuscules per genotype or treatment using the Zeiss confocal software blue edition ZEN2.0.

### RNA Analysis

RNA extraction was performed with Trisure (Bioline, Luckenwalde, Germany) or peqGold Trifast (Peqlab, Erlangen, Germany) according to the manufacturer’s protocol. Reverse transcription was performed with the Qiagen Omniscript RT Kit according to the manual (Qiagen, Hilden, Germany). Oligo(dT) primers served for the initial reverse transcription reaction on approximately 1 μg of total RNA after digestion with RNase-free DNase (Qiagen).

Aliquots of 0.2 μl of the 10 μl RT-reaction were used for the subsequent PCR reaction in the presence of SYBR Green with HotGoldStar DNA-Polymerase (Eurogentec, Seraing, Belgium) in a BioRad Cycler using the CFX System software based on the following program: denaturation at 95°C for 30 s, annealing for 30 s at 61°C and elongation for 30 s at 72°C, in a program of 45 cycles in 10 μl reaction volume. Primers (sequences in Supplementary Table S1) were designed to obtain a 50–150 bp amplicon with the help of the Primer3 software^2^. Melting curve analysis revealed amplification of one single amplicon, respectively. RNA accumulation analyses were performed with 2–3 technical replicates and 3–5 biological replicates for each measurement. Normalized expression levels were calculated with ubiquitin, tubulin and EF1α as reference genes (Supplementary Table S1) based on the following formula:

$$\mathrm{Normalized}\;\mathrm{expression}{\;}_{\mathrm{sample}\;\;(\mathrm{GOI})}\;=\;\frac{\mathrm{RQ}\;(\mathrm{GOI})}{{\left(\mathrm{RQ}\;\mathrm{sample}\;(\mathrm{Ref1})\;\times \;\mathrm{RQ}\;\mathrm{sample}\;(\mathrm{Ref2})\;\times \;\mathrm{RQ}\;\mathrm{sample}\;(\mathrm{Ref3})\right)}^{\mathrm{1/n}}}\;\;\;\;\;\;\;\;\;\;\;\;(1)$$

With RQ = relative quantity of a sample; Ref = reference target; GOI = gene of interest; *n* = number of reference targets. The average expression stability (*M*-value) of tubulin and ubiquitin references was 0.3955.

### Vascular Staining

Thin hand-cut sections were first incubated in safranin solution (1 g safranin diluted in 100 ml de-ionized water), de-stained with ethanol-acetic acid (100 ml 70% ethanol + 0.5 ml HCl) and subsequently stained with astrablue (0.5 g astrablue (Omikron, Reitberg) in 100 ml de-ionized water).

### Element Analysis via ICP-OE Spectrometry

For the analysis via ICP-OES (inductively coupled plasma optical emission spectrometry), the samples were completely dried at 80°C and pestled. 0.5 g sample were mixed with 5 ml 65% HNO_3_ and 3 ml 30% H_2_O_2_. The digestion in the microvave MarsXpress (CEM Corporation) and subsequent steps were carried out according to the instructions of the Gemeinschaftslabor Analytik (GLA) des Albrecht-Daniel-Thaer-Instituts für Agrar – und Gartenbauwissenschaften der HU Berlin.

The ICP-OES was performed with the iCAP 6300 Duo MFC (Thermo Fisher Scientific) by Dr. Kirsten Weiss (GLA). The analyzed elements were Ca (317.9 nm), Fe (259.9 nm), K (766.4 nm), Mn (257.6 nm), Zn (213.8 nm), and P (213.6 nm).

### Sterol Analysis of Microsomal Membranes

The isolation of the microsomal fraction was performed according to Larsson (1985). Fifty gram leaves of 6 weeks old tobacco plants were used for the experiment. Isolated microsomal fractions were stored at -20°C. The chloroform-methanol-extraction was performed according to the following protocol for phase separation. One volume of the sample was mixed with two volumes of chloroform/methanol (2:1), mixed properly and centrifuged 5 min at 2000 rpm at room temperature. The lower phase (containing the lipids) was collected and the rest was re-extracted as before. Both lower phases were pooled, stored at room temperature and shipped to the University Bonn. The subsequent steps were undertaken by the lipidomics platform^3^ headed by Peter Dörmann as described in Wewer et al. (2011).

### Statistics

To test whether the individuals of every single transgenic line differ significantly in their expression levels from the WT, the *t*-test with a cutoff level of α = 0.05 was used. To investigate whether phenotypical traits differ between plant genotypes and treatments factorial ANOVA was carried in accordance with the experimental design and means were compared by Tukey test as the *post hoc* procedure (α = 0.05).

## Results

### MSBP1 Affects Plant Development

The gene *SlMSBP1* codes for a 240 amino acid protein with one putative membrane spanning domain at its N-terminal domain and a conserved heme/steroid binding domain between position 71 and 167 (Supplementary Figure S1A). SlMSBP1 contains two conserved tyrosine-based ITAM activation motifs (YXX-aliphatic amino acid) at its C-terminal end, typically found twice at the cytoplasmic tail of cell surface proteins of the immune system (Abbas et al., 2015). In addition, two tyrosine-based sorting signals (YXXφ, φ = bulky, hydrophobic amino acid) are present, known for involvement in vesicle trafficking, endocytosis and cell sorting (Supplementary Figure S1B; Yang et al., 2008). Its topology predicts the N-terminus at the extracellular side of the membrane.

**Figure 1:**
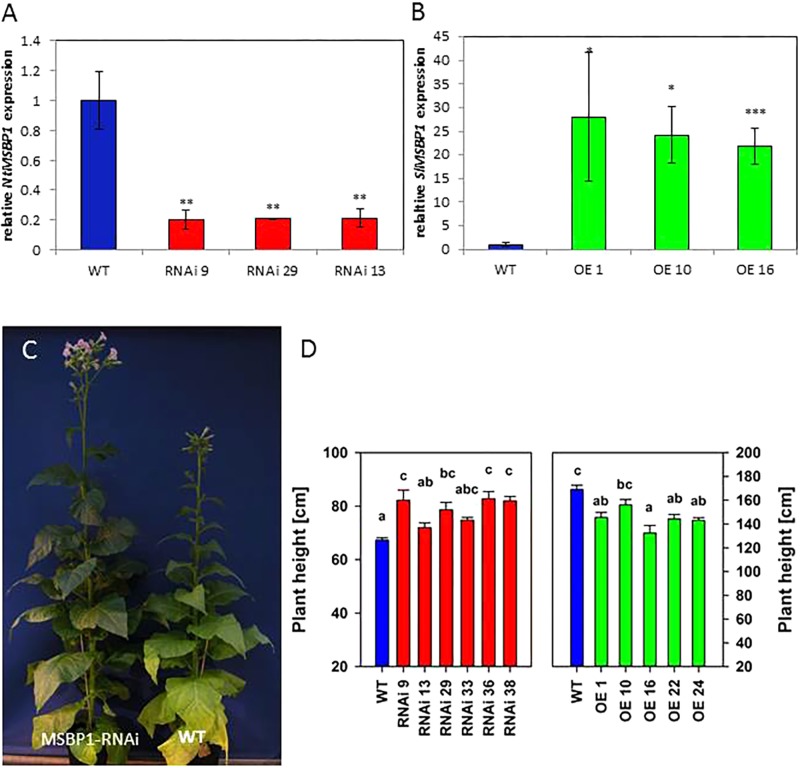
Silencing of *NtMSBP1* and overexpression of *MSBP1* in transgenic tobacco plants. **(A)** RNA accumulation analysis in source leaves of *NtMSBP1*-RNAi lines and wild type plants. *NtMSBP1* relative transcript levels were quantified by qPCR using tobacco genes encoding the translation factor EF1-α and ubiquitin for calibration. The relative expression values of RNAi-plants were normalized to the value obtained in WT plants (= 1) with ^∗∗^*P* < 0.01. **(B)** *SlMSBP1* expression in *MSBP1* overexpressing lines and WT plants. In case of *MSBP1*-overexpressing plants *SlMSBP1*-specific primers were used. Mean values and SEM are shown for 3–5 biological replicates (with ^∗^*p* < 0.05, ^∗∗∗^*p* < 0.001). **(C)** Tobacco *NtMBSP1*-RNAi line #9 (left) and wild type plant (right). **(D)** Heights of *NtMBSP1*-RNAi lines (left), *MSBP1* overexpressing lines (right) and wild type plants. Mean values and standard deviations are shown for 5–8 replicates (transgenic lines) or 16–28 replicates (wild type). Significant differences between values of the RNAi lines and wild type plants are indicated by different letters according to one way ANOVA with *post hoc* Tukey test and the SE is given. Differences in total plant height in the two experiments are due to differences in pot size and growth conditions.

In order to avoid co-suppression during overexpression of the gene *SlMSBP1*, tobacco was chosen as a close relative of tomato. Transgenic tobacco plants with reduced or increased *MSBP1* expression were generated and analyzed regarding size, leaf morphology, xylem proliferation, gene expression, flowering, and sugar accumulation. Quantitative qPCR revealed successful down-regulation of *NtMSBP1* expression in RNAi plants and overexpression of *SlMSBP1* in overexpressing (OE) lines (Figure 1A,B).

**Figure 2:**
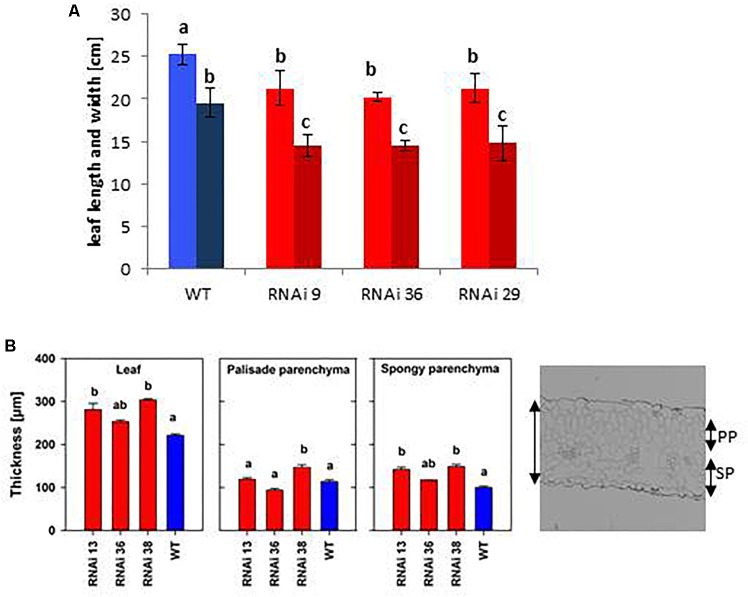
Leaf morphological parameters of *NtMSBP1*-RNAi tobacco plants. **(A)** Leaf length (light colored) and width (dark colored) and **(B)** total thickness of leaves, of palisade (PP) and spongy parenchyma (SP) are shown. Mean values and standard deviations of wild type plants and *MSBP1*-RNAi lines are presented for 4–5 replicates. Significant differences between values of the RNAi lines and wild type plants are indicated by different letters according to one way ANOVA.

**Figure 3:**
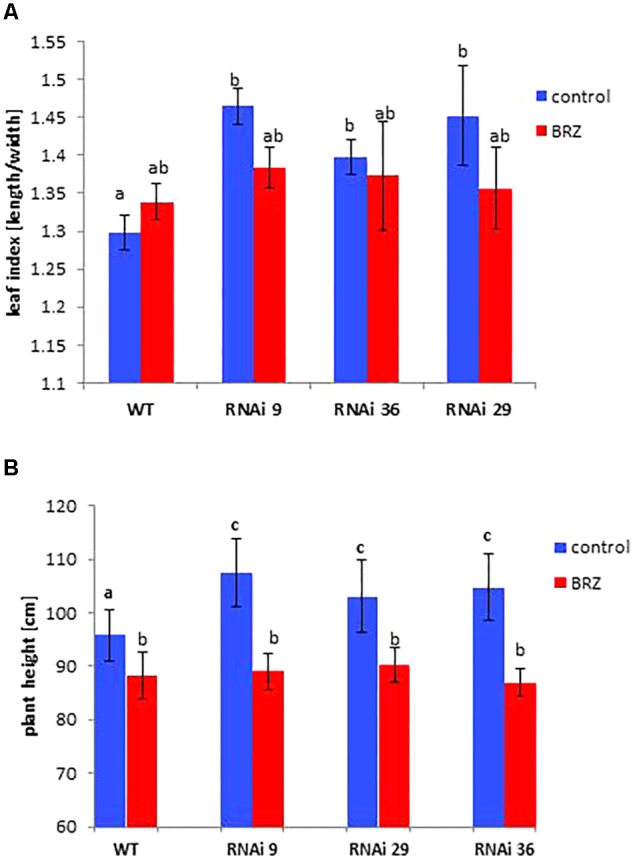
Rescue of the phenotypic changes of *NtMSBP1*-RNAi tobacco plants. **(A)** Shown are mean values and standard deviations of the ratios between leaf length and width (leaf index) and of **(B)** total plant of WT plants and *MSBP1*-RNAi tobacco lines 4–6 replicates). Leaves were locally treated with 1 μM BRZ or not. Significant differences according to a one-way ANOVA are indicated by different letters (α= 0.05).

Transgenic *MSBP1*-RNAi tobacco were significantly higher than tobacco WT plants (Figure 1C,D), whereas their leaf area (length and width of source leaves) was smaller (Figure 2A). The leaf length/width ratio in *NtMSBP1*-RNAi plants shifted significantly toward leaf length (Figure 3A) and, the specific leaf area (SLA = m^2^ kg^-1^ DW) was decreased (Supplementary Figure S2A). This was accompanied by thicker leaves resulting from a thicker layer of the spongy parenchyma (Figure 2B). Inverse phenotypical traits were observed for the *MSBP1*-OE lines. Total plant size decreased compared to wild type plants (Figure 1D) and the specific leaf area (SLA) increased (Supplementary Figure S2B) implying the development of thinner leaves.

### MSBP1 as Inhibitor of BR Signaling

In order to test the hypothesis that MSBP1 acts as an inhibitor of brassinosteroid (BR) signaling, tobacco wild type and *MSBP1*-RNAi plants were sprayed every other day with brassinazole (BRZ), an inhibitor of BR biosynthesis. BRZ treatment diminished the differences in plant height, and leaf index between tobacco WT and *NtMSBP1*-RNAi plants (Figure 3).

Since brassinosteroids are described to promote Arabidopsis flowering (Li et al., 2010), flowering behavior was investigated in *MSBP1*-RNAi and OE lines and phenotypes were tried to be rescued by treatments with the synthetic BR epi-brassinolide (epi-BL). BRZ treatment was also included in the experiment. BRZ treatments delayed flowering of wild type plants by several days (Supplementary Figure S3). However, the onset of flowering of *MSBP1*-OE lines was not responding to the BRZ treatment. In addition, they were rather insensitive to epi-BL (Supplementary Figure S4). In contrast, flowering of *MSBP1*-RNAi plants was sensitive to epi-BL treatment (Supplementary Figure S5), i.e., they blossomed earlier than wild type plants under similar conditions.

BR deficient Arabidopsis mutants are characterized by xylem under-proliferation (Szekeres et al., 1996), whereas a BRZ treatment inhibits secondary xylem development (Nagata et al., 2001). Consistently, a higher number of xylem vessels in vascular bundles of *MSBP1*-RNAi plants was visually detected (Supplementary Figure S6), but quantification of the total xylem area in cross-sections revealed no significant differences between tobacco WT and transgenic lines.

### MSBP1 Affects Mycorrhiza Functioning

MSBP1 is known for its regulatory function of AM colonization in *M. truncatula* (Kuhn et al., 2010). In addition, a BR biosynthesis mutant showed reduced colonization by AM fungi (Bitterlich et al., 2014a). Therefore, the role of MSBP1 in the mycorrhizal symbiosis was investigated. Inoculation of *MSBP1*-RNAi and –OE tobacco lines with the AM fungus *Rhizoglomus irregularis* was performed. Mycorrhizal parameters were estimated after staining, and the activity of the fungus was quantified by qPCR of fungus-specific marker genes.

Root infection frequency (F), hyphal spread inside the roots (M) and arbuscule abundance (A) were not different in *NtMSBP1*-RNAi lines compared to the wild type (data not shown). In agreement with these data, the mRNA quantification of the AM fungus-specific marker gene *RiGAPDH* in colonized roots of *NtMSBP1*-RNAi plants revealed no significant changes in the transcript amount in most of the transgenic lines (Supplementary Figure S7). Mycorrhizal parameters and fungal activity were also quantified in *MSBP1*-OE lines. Statistical evaluation of the degree of colonization by staining and of fungal activity as determined by the level of *RiGAPDH* transcript quantification did not show a significant effects taking all *MSBP1*-overexpressing lines into account (Supplementary Figure S7).

**Figure 4:**
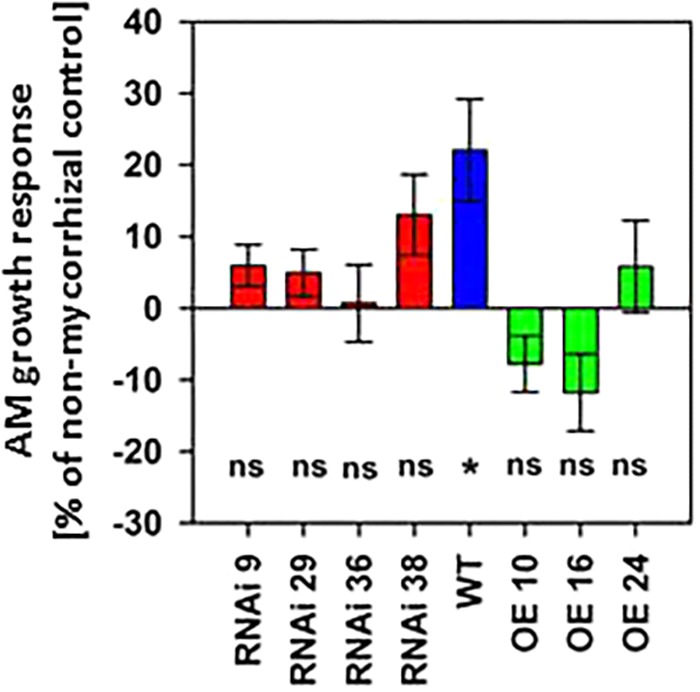
Total biomass production of *NtMSBP1*-RNAi and *MSBP1*-overexpressing tobacco lines compared to non-mycorrhizal control plants. Mean values and standard deviations are shown for six replicates of mycorrhizal and non-mycorrhizal wild type plants and of *NtMSBP1-RNAi* and *MSBP1*-overexpressing lines. Whether the growth AM response is different from zero was tested by ANOVA (*n* = 6, *P* < 0.05).

While a positive effect of mycorrhization on biomass production of wild type plants was observed, this effect disappeared in RNAi lines, and *MSBP1*-OE lines rather showed a decrease in total biomass production upon mycorrhization (Figure 4 and Supplementary Figure S10A). Regarding internode elongation (Supplementary Figure S10B), the MSBP1-overexpressing plants also do not show a positive response upon mycorrhization and were insensitive toward epi-BL treatment. Differences in the total biomass between inoculated and non-inoculated plants (shown in Figure 4 and Supplementary Figure S10A) depend on the level of *MSBP1*-transcripts analyzed in the root tissue of the plants from the same experiment (shown in Supplementary Figure S8). *MSBP1* overexpression resulted in a decrease in biomass development after mycorrhization of the corresponding lines (Figure 4 and Supplementary Figure S10A).

**Figure 5:**
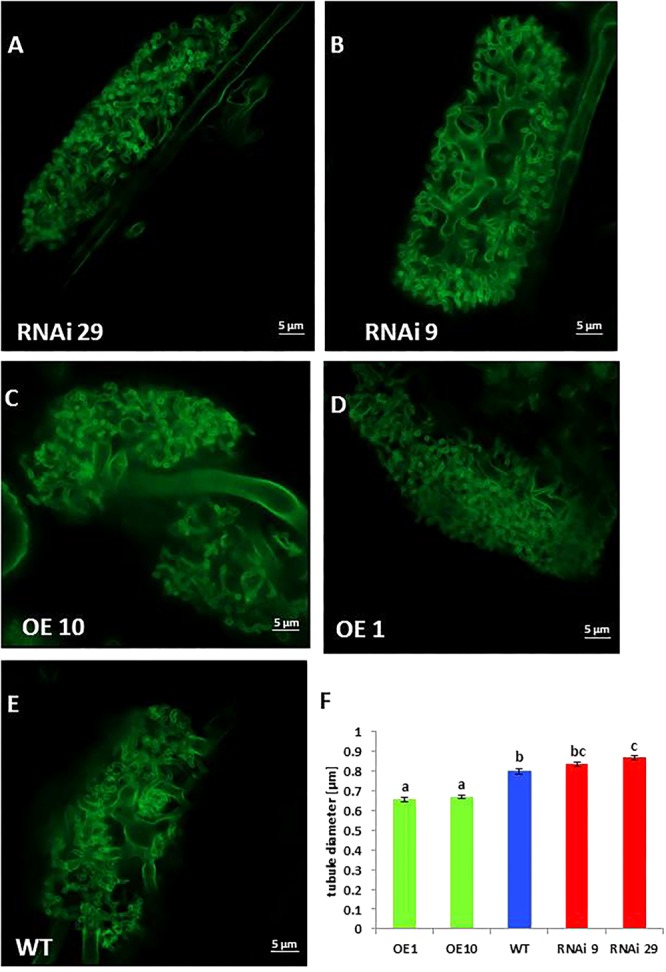
Arbuscule morphology of *NtMSBP1*-RNAi **(A,B)**, *MSBP1*-overexpressing **(C,D)** or wild type **(E)** tobacco lines**.** Roots were stained with WGA-FITC and analyzed by confocal microscopy. Measurement of tubule diameters was performed using the ZEN2.0 confocal software **(F)**. Mean values and standard deviations of at least 20 different tubules from 10 different arbuscules for each genotype are shown and the SEM is given. Significant differences according to a one-way ANOVA are indicated by different letters with *P* < 0.001. Scale bars are 5 μm in length.

Further analysis of mycorrhization was performed by staining fungal structures using WGA-FITC conjugate (Figure 5). This analysis revealed differences in arbuscular morphology between roots of wild type plants, *MSBP1*-RNAi and *MSBP1*-OE overexpression lines (Figure 5). The diameter of the finest tubular branches of stained arbuscules (for details see schematic drawing in Supplementary Figure S11) was determined by help of the confocal software ZEN 2.0. Compared to the wild type, the tubule diameter was significantly decreased in two *MSBP1*-overexpressing lines and consistently increased only in RNAi lines (Figure 5C–E). In order to test, if tubular diameter had an impact on the nutrient transfer in mycorrhizal plants, a multi-element analysis was carried out. This revealed that the concentration of phosphorus, iron and zinc is increased only in *MSBP1*- transgenic plants in response to mycorrhization, whereas WT showed lower amounts (Supplementary Figure S12). Regarding the accumulation of mineral elements such as manganese, and calcium in the above-ground parts of mycorrhizal plants, only *MSBP1*-RNAi plants show higher contents than non-mycorrhizal control plants and significantly higher content of phosphate, iron, manganese and calcium than mycorrhizal WT plants (Supplementary Figure S12). Thus, *MBSP1*-silencing seems to improve plant nutrition via the mycorrhizal pathway.

Regarding the membrane sterol content of roots, the amount of cholesterol incorporated into membranes is slightly decreased in both, MSBP1-silenced as well as MSBP1-overexpressing plants (Supplementary Figure S13). Thus it is unlikely that the sterol mass fraction is responsible for altered arbuscular architecture.

### Effects of BRs on Mycorrhizal Tobacco and Tomato WT Plants

**Figure 6:**
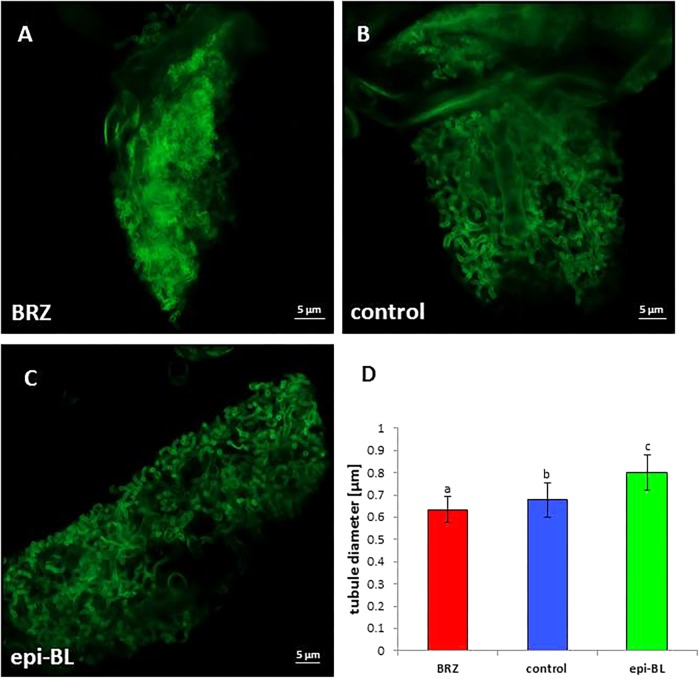
Arbuscule morphology in tobacco wild type roots. Tobacco mycorrhiza treated with brassinazole **(A)**, with DMSO as control **(B),** or with epi-brassinolide **(C)**. Roots were stained with WGA-FITC and analyzed by CLSM. **(D)** Tubule diameter estimation determined with the ZEN 2.0 software. Mean values and SE are shown for 20–80 tubular branches from 15 different arbuscule replicates for each treatment. Significant differences between DMSO control and BRZ or epi-BL treatment were calculated by a one-way ANOVA and indicated by different letters (with *P* < 0.05). Scale bars are 5 μm in length.

Since expression levels of *MSBP1* affect the mycorrhizal symbiosis and MSBP1 is assumed to inhibit BR signaling, it was tested whether similar effects could be achieved using BR or BR inhibitors. Tomato and tobacco WT plants inoculated or not with the AM fungus *R. irregularis* were treated with epi-BL or BRZ and analyzed. Arbuscules showed the same morphological effect, when BRZ was applied as previously seen in *MSBP1*-OE lines: arbuscules appear highly branched with a bushy appearance (Figure 6). Quantification of tubule diameter of arbuscular branches revealed significantly smaller values after BRZ treatment than for the untreated control (Figure 6D). Interestingly, epi-BL-treatment resulted in even larger values for arbuscular tubule diameters (Figure 6D).

**Figure 7:**
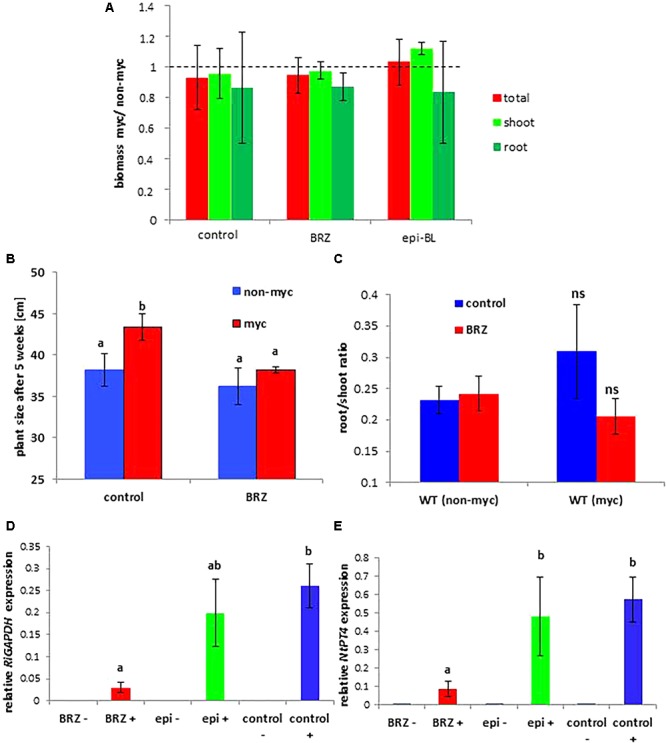
Mycorrhization parameters as influenced by brassinazole (BRZ) and epi-brassinolide (epi-BL) treatment in tobacco WT plants. **(A)** Shown are mean values and standard deviations of mycorrhiza-induced increase in **(A)** plant biomass of five replicates of wild type tobacco plants treated or not with brassinazole (BRZ) or with epi-brassinolide (epi-BL). Although biomass parameters do not reveal significant changes, a biomass increase by mycorrhization is only observed in epi-BL treated plants. Tobacco WT plants were harvested after 9 weeks of colonization. **(B)** Mycorrhiza-induced increase in plant size is only observed in control plants, whereas the gain in biomass by mycorrhization is abolished by BRZ treatment. **(C)** root/shoot ratios of three replicates WT tobacco plants treated with or without BRZ. Tobacco WT plants in **(B,C)** were harvested 5 weeks after inoculation with *R. irregularis*. Results were highly reproducible in different experiments. **(D, E)** Quantification of fungal *RiGAPDH* (left) and tobacco *NtPT4* (left) transcripts using tubulin as reference genes. Mean values and SEM are shown for three biological and two technical replicates. RNA accumulation values of *RiGAPDH* in non-mycorrhizal plants were below the detection level and are therefore not shown. One-way ANOVA with α= 0.05 reveals significant differences indicated by different letters.

Tobacco WT plants treated with epi-BL showed the largest mycorrhiza-induced increases in biomass production in AM plants compared to non-treated or BRZ-treated plants (Figure 7A–C). These results were reproducible in different experiments where plants were harvested either 9 weeks (Figure 7A) or 5 weeks after inoculation with *R. irregularis* with more prominent differences after 5 weeks (Figure 7B,C). The amount of fungal-specific transcripts for *RiGAPDH* was also reduced in BRZ-irrigated roots (Figure 7D). Up-regulation of the plant phosphate transporter *NtPT4* expression argues for functionality of arbuscular structures in tobacco WT plants (Figure 7D).

Tomato WT plants showed a mycorrhiza-induced increase in fresh weights when treated with DMSO (control) or with BRZ (Supplementary Figure S14). In the presence of exogenously applied epi-BL this increase in fresh biomass after mycorrhization was even higher (Supplementary Figure S14A). Quantification of fungal transcripts (*RiGAPDH*) and of the transcript of the arbuscule specific phosphate transporter gene *PT4* confirmed successful root colonization in all cases. Gain in biomass is highest in epi-BL treated plants although the expression of *RiGAPDH* and *SlPT4* is higher in BRZ treated samples (Supplementary Figure S14). Plant growth responses of epi-BL treated mycorrhizal tomato and tobacco WT plants are therefore comparable.

Thus, *MSBP1*-inhibition in transgenic tobacco plants causes similar effects like epi-BL treatment of tobacco and tomato WT plants in terms of mycorrhization indicating that the effect of MSBP1 on arbuscular morphology might be via its inhibitory function on BR signaling.

## Discussion

### MSBP1 as Inhibitor of BR Signaling

The first hypothesis of the current study stated that MSBP1 is an inhibitor of brassinosteroid (BR) signaling also in tobacco as previously postulated for *AtMSBP1* in Arabidopsis thaliana (Song et al., 2009). This hypothesis was tested with the help of transgenic tobacco plants where it was investigated, if up- or down-regulation of MSBP1 expression affects leaf morphology, plant height and flowering behavior (Figure 1, 2 and Supplementary Figures S3–S5). As previously observed in *dim1* mutants defective in BR biosynthesis, leaf thickness is affected also in *MSBP1*-silenced plants: the leaves of *MSBP1*-RNAi plants are thicker, leading to a reduced SLA, whereas the opposite can be observed in *MSBP1*-overexpressors. This is in agreement with a potential role of MSBP1 as an inhibitor of BR signaling in tobacco. Rescue experiments in previous studies were successfully used to restore the dwarfed phenotype of BR-defective mutants (Azpiroz et al., 1998). Therefore, we aimed at restoration of the MSBP1 phenotype by application of the BR inhibitor brassinazole (BRZ). The fact that BRZ treatment restored the WT phenotype of plant and leaf morphology in *MSBP1*-RNAi plants (Figure 3) additionally argues for an inhibitory role of MSBP1 in BR signaling.

The hypersensitivity of *MSBP1*-inhibited plants and the rather insensitive behavior of *MSBP1*-overexpressing plants of flower induction in response to the BR epi-brassinolide (epi-BL; Supplementary Figures S3–S5) treatment further points to a negative role of MSBP1 in BR signal perception and transduction. Plant size, flowering behavior, xylem proliferation, BR responses of *MSBP1*-silenced and –overexpressing plants further supports the assumption that MSBP1 acts as a negative BR signaling component.

### BRs Affect Mycorrhization and Arbuscular Morphology

Our previous investigations revealed reduced arbuscular abundance and mycorrhization intensity of mutants defective in BR biosynthesis. The tomato *dx* mutant as well as the rice *brd2-1* mutant which both are deficient in the DWF1 sterol reductase show reduced mycorrhization intensity if inoculated with *Rhizoglomus irregularis* (Bitterlich et al., 2014a,b). Therefore, an impact of brassinosteroids on mycorrhization can be expected if supplied directly to the root system of mycorrhizal plants.

Although experiments with tobacco wild type plants externally treated with epi-BL do not show significant effects on the amount of fungus-specific RiGAPDH and of the mycorrhiza-induced plant phosphate transporter PT4 transcripts (Figure 7D),a positive effect of epi-BL on the biomass production of mycorrhizal tobacco and tomato WT plants was observed (Figure 7 and Supplementary Figure S14A). In parallel, arbuscular architecture in WT roots changed when plants were treated externally treated with BRZ or epi-BL (Figure 6). Epi-BL treated roots showed arbuscules with higher tubular diameters, whereas BRZ treated roots harbored arbuscules with smaller tubular diameters than the corresponding mock control (Figure 6).

If MSBP1 is indeed active as an inhibitor of brassinosteroid signaling, then inhibition of BR signaling in MSBP1 overexpressing plants could have similar effects on mycorrhization as observed in case of inhibition of BR biosynthesis by BRZ application. In agreement with this hypothesis, a very similar phenomenon was observed in transgenic MSBP1 tobacco plants, that is, inhibition of MSBP1 expression resulting in higher tubular diameter of arbuscules, whereas overexpression of the putative inhibitor of BR signaling led to smaller tubules (Figure 5, 6).

A reduction of the diameter of tubular branches and highly branched arbuscules with “bushy” appearance would lead to an increase of the interface surface between the host plant and the fungus. Consequently, this could increase exchange activities between both symbiotic partners where the fungus receives carbon containing compounds such as sugars and lipids (Siebers et al., 2016; Bravo et al., 2017), whereas the plant obtains mineral nutrients (Garcia et al., 2016). Against our expectation, the increase in the surface area between plant and fungus is not leading to increased uptake of mineral elements. In contrast, the P, Mn, Fe, Zn, and Ca contents in colonized plants are significantly higher in MSBP1-RNAi plants with arbuscules showing a higher diameter of tubules than in MSBP1-overexpressing or WT plants (Supplementary Figure S12). This did, however, not lead to an increased mycorrhizal effect as this effect disappeared also in lines with down-regulated *MSBP1* expression (Figure 4). The reasons for that merit further investigation and may relate to a counter-acting increased transfer of carbohydrates to the fungus, or else. The activity of *R. irregularis*, however, was not affected by manipulation of *MSBP1* expression as revealed by qPCR and ink staining of colonized roots (Supplementary Figures S9, S11B).

From recent investigations it became clear that within AM symbiosis the plant partner defines the tubule architecture where the fungus adapts to Lanfranco et al. (2016). Arbuscule morphology is known to be affected when H^+^-ATPases or ABC-transporter expression is disturbed (Zhang et al., 2010; Krajinski et al., 2014) leading in most cases to truncated or aborted arbuscules. Nothing is described so far regarding effects on the ramification morphology of arbuscular branches. It should be investigated in future experiments whether the branching of fungal arbuscules and the curvature of the periarbuscular membrane is related to the biophysical properties of the membrane and how this is crucial for mycorrhizal functioning.

### MSBP1 Has Multiple Functions

As mentioned above MSBP1 is assumed to act as inhibitor of BR signaling and analysis of MSBP1-silenced and – overexpressing plants is consistent with this assumption. In this case it is expected that down-regulation of MSBP1 positively impacts mycorrhization since BRs have a positive effect on mycorrhization in tobacco and tomato WT plants.

MSBP1 might fulfill a dual function by inhibiting BR signaling on the one side, and enhancing vesicle trafficking and endocytosis of membrane proteins on the other (Yang et al., 2008; Song et al., 2009). Also its negative effect on BR signaling was previously explained by enhancement of endocytosis of the BR co-receptor BAK1 (Song et al., 2009).

For SlSUT2, a sucrose retrieval carrier at the periarbuscular membrane, enhanced endocytosis is assumed to impact root colonization by AM fungi (Bitterlich et al., 2014a,b). Sucrose supply to the fungus and BR-dependent effects might have two opposite effects of MSBP1 on root colonization. And the factors determining which of both effects predominates are still unknown. Interestingly, the expression of sucrose transporter SlSUT2 is upregulated in response to biotic interactions, whereas MSBP1 expression is rather down-regulated (Supplementary Figures S8A, S15) according to the TomExpress database, supporting this hypothesis.

Differences between *Medicago truncatula* and the solanaceous host plants might be due to a shift of this equilibrium to one or the other side. Alternatively, these differences can be due to species-dependent differences or the fact that silencing of *MSBP1* in Medicago was limited locally to the root system only (Kuhn et al., 2010).

### MSBP1 Affects Sterol Content and Membrane Composition

MSBP1 seems also to impact the sterol composition of the membrane since both, the overexpressing as well as the RNAi plants showed a slightly decreased cholesterol content (Supplementary Figure S13). In previous studies, it was discussed whether MSPB1 affects AM colonization via regulation of the sterol homeostasis and interferes with microdomain assembly (Bapaume and Reinhardt, 2012). In Arabidopsis, AtMSBP1 is able to bind to progesterone, testosterone and epi-brassinolide *in vitro*, but also to stigmasterol (Yang et al., 2008), which is part of the plasma membrane and is often distributed asymmetrically in the two leaflets of the plasma membrane. Phytosterols are known to be enriched in membrane microdomains and affect the fluidity and dynamics of the plasma membrane. Membrane microdomains are considered as signaling platforms, which are not only involved in symbiotic but also in pathogenic plant interactions (Bhat et al., 2005; Keinath et al., 2010).

Further investigations are necessary to answer the question whether the multiple MSBP1 effects on vesicle trafficking, endocytosis, arbuscular morphology, and membrane composition are based on the ability of MSBP1 to bind to sterol components.

## Conclusion

• Brassinosteroids affect the morphology of arbuscular branches, the nutrient content of colonized host plants and the biomass production in response to mycorrhization. Treatment with the inhibitor BRZ had a negative effect on fungus-specific gene expression. Therefore, a fine-tuning role of BRs in the AM symbiosis is suggested.
• Effects of up- or down-regulation of *MSBP1* in transgenic tobacco plants are consistent with an inhibitory role of MSBP1 in BR signaling. The *MSBP1*-overexpression phenotype can be mimicked by inhibition of BR synthesis via BRZ.
• MSBP1 potentially affects mycorrhization via its inhibitory role in BR signaling. A balanced expression of the protein is therefore important for a positive outcome of the symbiosis.

## Data Availability

All datasets generated for this study are included in the manuscript and/or the Supplementary Files.

## Author Contributions

CK planned and designed the research. BJ, HJ, LvS, and MR performed the experiments. LvS, MB, PF, and CK analyzed and interpreted the data. PF and CK wrote the manuscript.

## Conflict of Interest Statement

The authors declare that the research was conducted in the absence of any commercial or financial relationships that could be construed as a potential conflict of interest.

## Footnotes

1 www2.dijon.inra.fr/mychintec/Mycocalc-prg/download.html

2 http://bioinfo.ut.ee/primer3-0.4.0/

3 https://www.imbio.uni-bonn.de/molekulare-biotechnologie/lipidomics-platform
