# Supplementary material for: Brassinosteroids Affect the Symbiosis Between the AM Fungus Rhizoglomus irregularis and Solanaceous Host Plants
Source: Front Plant Sci. 2019 May 15;10:571. doi: 10.3389/fpls.2019.00571 (PMC6530493; doi:10.3389/fpls.2019.00571)
Supplement: Supplementary file 1 [file Table_1.DOCX]

**Supplementary Table 1**

Primer pairs for RNA accumulation analyses of *Nicotiana tabacum* genes by qRT-PCR:

| **Name of the gene** | **Forward primer** | **Reverse primer** |
| --- | --- | --- |
| NtMSBP1 | gttgttgctttggctcttgc | aggctgcatctgttcctcaa |
| NtPT4 | cgtttattgctgcggtgttt | gctccgtggacaaaatttca |

Primer pairs for tobacco reference genes according to Schmidt & Delaney ([Schmidt and Delaney, 2010](#_ENREF_25)):

| **Name of the gene** | **Forward primer** | **Reverse primer** |
| --- | --- | --- |
| Actin (Tac9) | CCTGAGGTCCTTTTCCAACCA | GGATTCCGGCAGCTTCCATT |
| Elongation factor 1a (EF-1a) | TGAGATGCACCACGAAGCTC | CCAACATTGTCACCAGGAAGTG |
| α-Tubulin (tubA1) | CAAGACTAAGCGTACCATCCA | TTGAATCCAGTAGGGCACCAG |
| β-Tubulin | GCATCTTTGCGTACACTTTGCT | ACATAAGCCCAAAACTAGCTGGA |
| Ubiquitin-conjugating enzyme E2 (Ntubc2) | CTGGACAGCAGACTGACATC | CAGGATAATTTGCTGTAACAGATTA |

Primer pairs for RNA accumulation analyses of *Solanum lycopersicum* genes by qRT-PCR:

| **Name of the gene** | **Forward primer** | **Reverse primer** |
| --- | --- | --- |
| SlMSBP1 | GGACCTGGAGGACCTTCTGC | AATGGACCAAGGCCAGAGAT |
| SlPT4 | agccccaggcagattatgtt | catgttaatcgcggcttgtt |
| SlTEF | TGG AAC TGT CCC TGT TGG TC | ACA TTG TCA CCA GGG AGT GC |

Primer pairs for RNA accumulation analyses of *Rhizoglomus irregularis* genes by qRT-PCR:

| **Name of the gene** | **Forward primer** | **Reverse primer** |
| --- | --- | --- |
| RiGAPDH | gacgtctcagttgttgattta | tttggcatcaaaaatactaga |

Primers for cloning of the MSBP1 sense construct:

| **Name of the primer** | **Primer sequence** |
| --- | --- |
| MSBP1-KpnI fw | GAGAGGTACCATGGCCCTACAACAATG |
| MSBP1-BamHI rev | GAGAGGATCCTTAGTCCTTCTTGTCGAC |
