# Supplementary material for: Brassinosteroids Affect the Symbiosis Between the AM Fungus Rhizoglomus irregularis and Solanaceous Host Plants
Source: Front Plant Sci. 2019 May 15;10:571. doi: 10.3389/fpls.2019.00571 (PMC6530493; doi:10.3389/fpls.2019.00571)
Supplement: Supplementary file 2 [file Presentation_1.PPTX]

## Slide 1
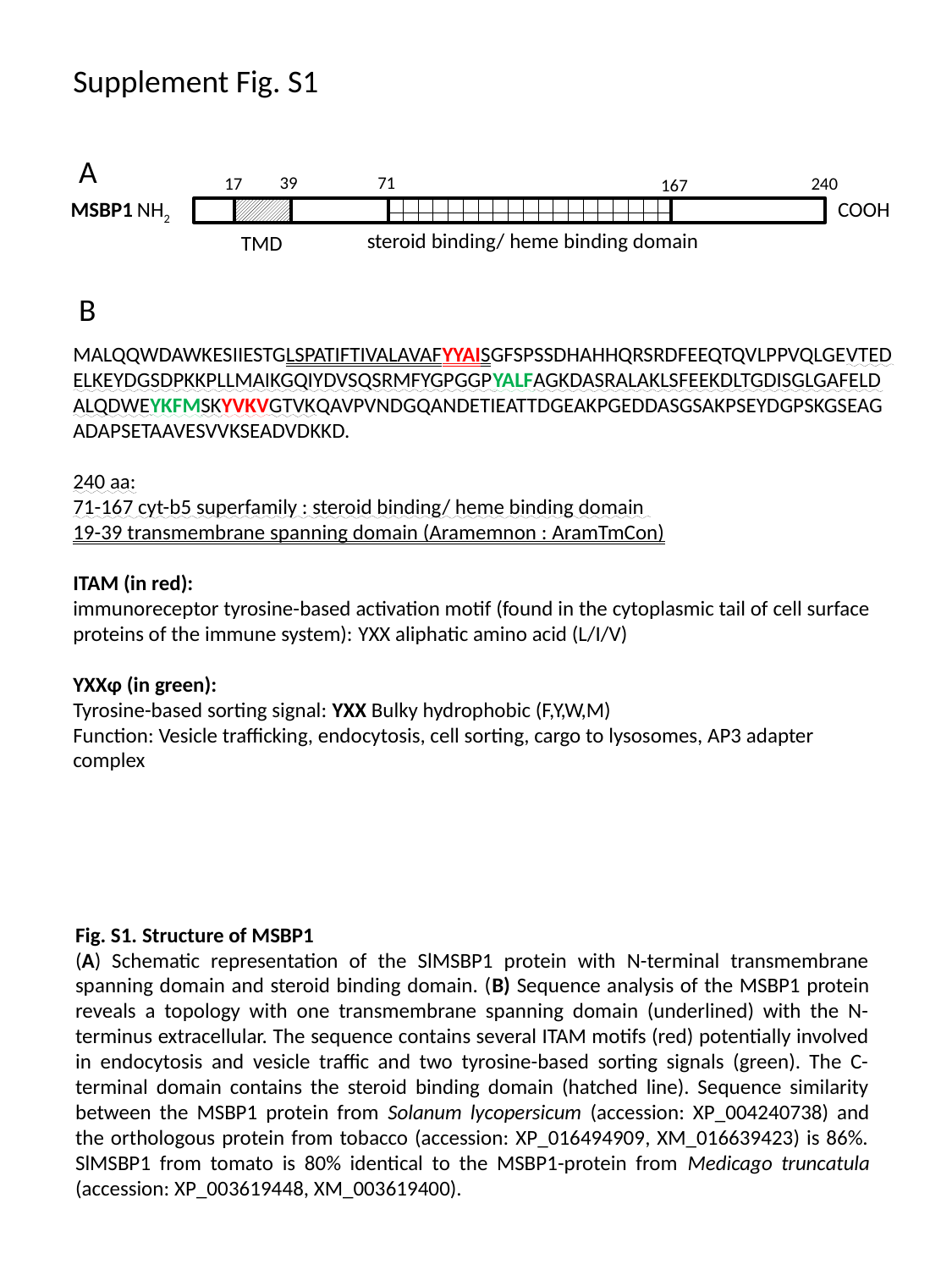

Supplement Fig. S1
A
39
71
17
240
167
COOH
MSBP1
NH2
steroid binding/ heme binding domain
TMD
B
MALQQWDAWKESIIESTGLSPATIFTIVALAVAFYYAISGFSPSSDHAHHQRSRDFEEQTQVLPPVQLGEVTEDELKEYDGSDPKKPLLMAIKGQIYDVSQSRMFYGPGGPYALFAGKDASRALAKLSFEEKDLTGDISGLGAFELDALQDWEYKFMSKYVKVGTVKQAVPVNDGQANDETIEATTDGEAKPGEDDASGSAKPSEYDGPSKGSEAGADAPSETAAVESVVKSEADVDKKD.
240 aa:
71-167 cyt-b5 superfamily : steroid binding/ heme binding domain
19-39 transmembrane spanning domain (Aramemnon : AramTmCon)
ITAM (in red):
immunoreceptor tyrosine-based activation motif (found in the cytoplasmic tail of cell surface proteins of the immune system): YXX aliphatic amino acid (L/I/V)
YXXφ (in green):
Tyrosine-based sorting signal: YXX Bulky hydrophobic (F,Y,W,M)
Function: Vesicle trafficking, endocytosis, cell sorting, cargo to lysosomes, AP3 adapter complex
Fig. S1. Structure of MSBP1
(A) Schematic representation of the SlMSBP1 protein with N-terminal transmembrane spanning domain and steroid binding domain. (B) Sequence analysis of the MSBP1 protein reveals a topology with one transmembrane spanning domain (underlined) with the N-terminus extracellular. The sequence contains several ITAM motifs (red) potentially involved in endocytosis and vesicle traffic and two tyrosine-based sorting signals (green). The C-terminal domain contains the steroid binding domain (hatched line). Sequence similarity between the MSBP1 protein from Solanum lycopersicum (accession: XP_004240738) and the orthologous protein from tobacco (accession: XP_016494909, XM_016639423) is 86%. SlMSBP1 from tomato is 80% identical to the MSBP1-protein from Medicago truncatula (accession: XP_003619448, XM_003619400).

## Slide 2
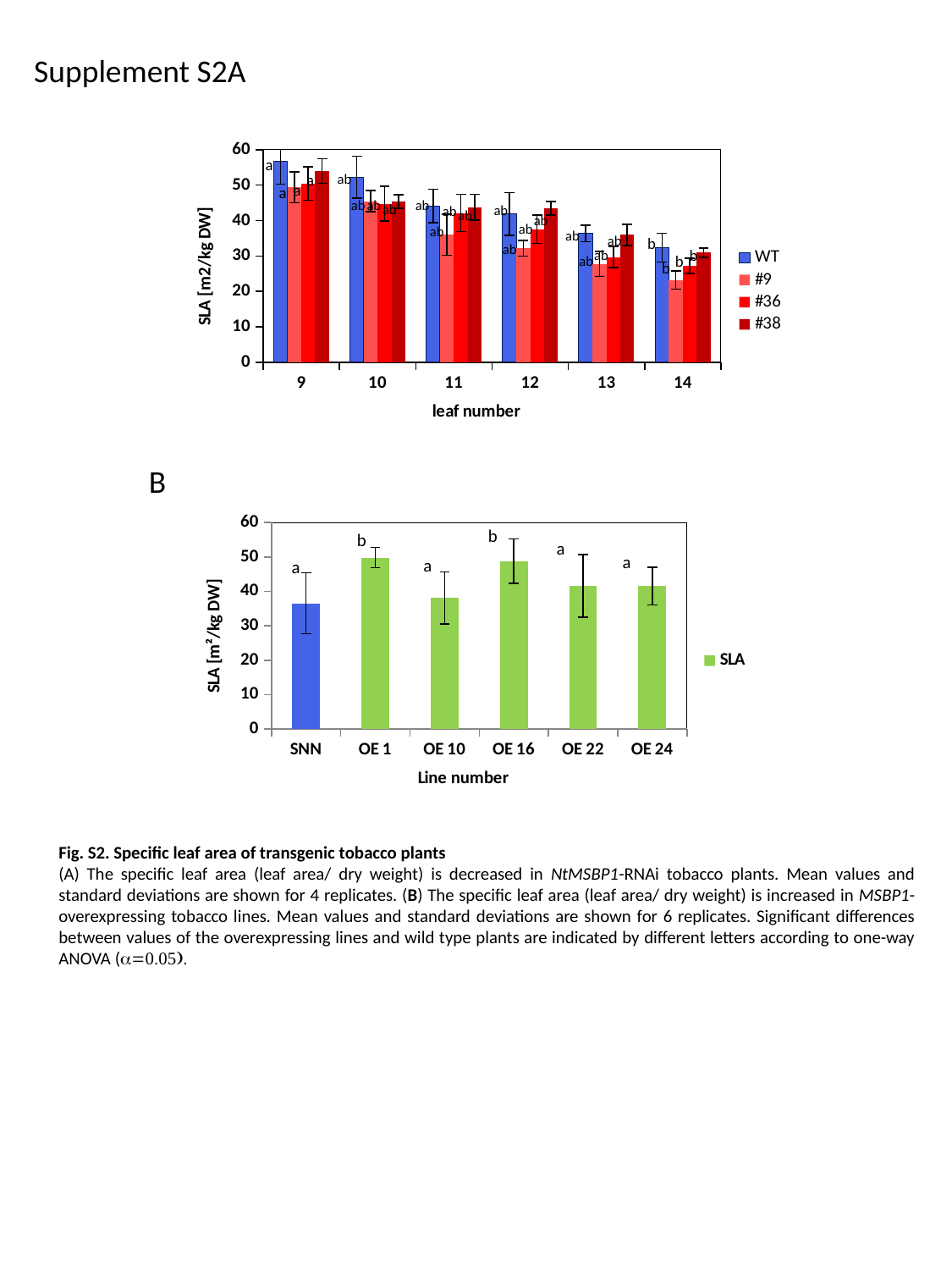

Supplement S2A
### Chart
| Category | | | | |
|---|---|---|---|---|
| 9 | 56.71311012990429 | 49.349570120881744 | 50.458761519843925 | 53.9962421152514 |
| 10 | 52.300064225565 | 45.49352315545497 | 44.76720842070493 | 45.35037658545449 |
| 11 | 44.14303923014541 | 36.01235545300108 | 42.15525176512563 | 43.721357214912835 |
| 12 | 41.890810017371 | 32.17619173950108 | 37.52625611108856 | 43.4813031021815 |
| 13 | 36.38581498492958 | 27.74350074228756 | 29.722024673966416 | 35.98306188953659 |
| 14 | 32.35295649001425 | 23.22705274850643 | 27.255502069157217 | 30.94129456389552 |a
ab
a
a
a
ab
ab
ab
ab
ab
ab
ab
ab
ab
ab
ab
ab
b
ab
b
ab
b
ab
b
B
### Chart
| Category | |
|---|---|
| SNN | 36.5272872324523 |
| OE 1 | 49.83255841474985 |
| OE 10 | 38.110587695458015 |
| OE 16 | 48.806445841047555 |
| OE 22 | 41.61707039713898 |
| OE 24 | 41.56866280017441 |b
b
a
a
a
a
Fig. S2. Specific leaf area of transgenic tobacco plants
(A) The specific leaf area (leaf area/ dry weight) is decreased in NtMSBP1-RNAi tobacco plants. Mean values and standard deviations are shown for 4 replicates. (B) The specific leaf area (leaf area/ dry weight) is increased in MSBP1-overexpressing tobacco lines. Mean values and standard deviations are shown for 6 replicates. Significant differences between values of the overexpressing lines and wild type plants are indicated by different letters according to one-way ANOVA (a=0.05).

## Slide 3
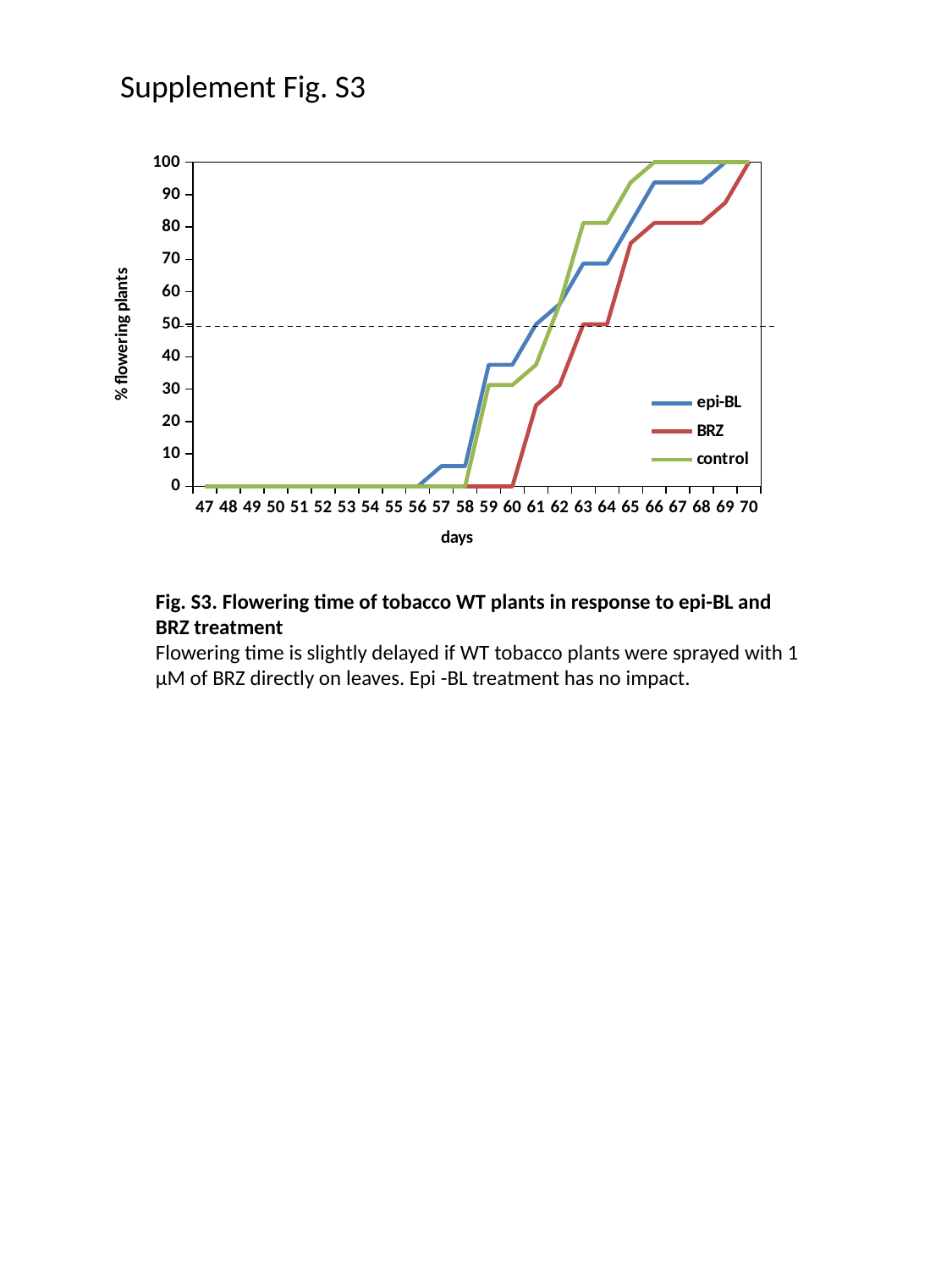

Supplement Fig. S3
### Chart
| Category | epi-BL | BRZ | control |
|---|---|---|---|
| 47 | 0.0 | 0.0 | 0.0 |
| 48 | 0.0 | 0.0 | 0.0 |
| 49 | 0.0 | 0.0 | 0.0 |
| 50 | 0.0 | 0.0 | 0.0 |
| 51 | 0.0 | 0.0 | 0.0 |
| 52 | 0.0 | 0.0 | 0.0 |
| 53 | 0.0 | 0.0 | 0.0 |
| 54 | 0.0 | 0.0 | 0.0 |
| 55 | 0.0 | 0.0 | 0.0 |
| 56 | 0.0 | 0.0 | 0.0 |
| 57 | 6.25 | 0.0 | 0.0 |
| 58 | 6.25 | 0.0 | 0.0 |
| 59 | 37.5 | 0.0 | 31.25 |
| 60 | 37.5 | 0.0 | 31.25 |
| 61 | 50.0 | 25.0 | 37.5 |
| 62 | 56.25 | 31.25 | 56.25 |
| 63 | 68.75 | 50.0 | 81.25 |
| 64 | 68.75 | 50.0 | 81.25 |
| 65 | 81.25 | 75.0 | 93.75 |
| 66 | 93.75 | 81.25 | 100.0 |
| 67 | 93.75 | 81.25 | 100.0 |
| 68 | 93.75 | 81.25 | 100.0 |
| 69 | 100.0 | 87.5 | 100.0 |
| 70 | 100.0 | 100.0 | 100.0 |Fig. S3. Flowering time of tobacco WT plants in response to epi-BL and BRZ treatment
Flowering time is slightly delayed if WT tobacco plants were sprayed with 1 µM of BRZ directly on leaves. Epi -BL treatment has no impact.

## Slide 4
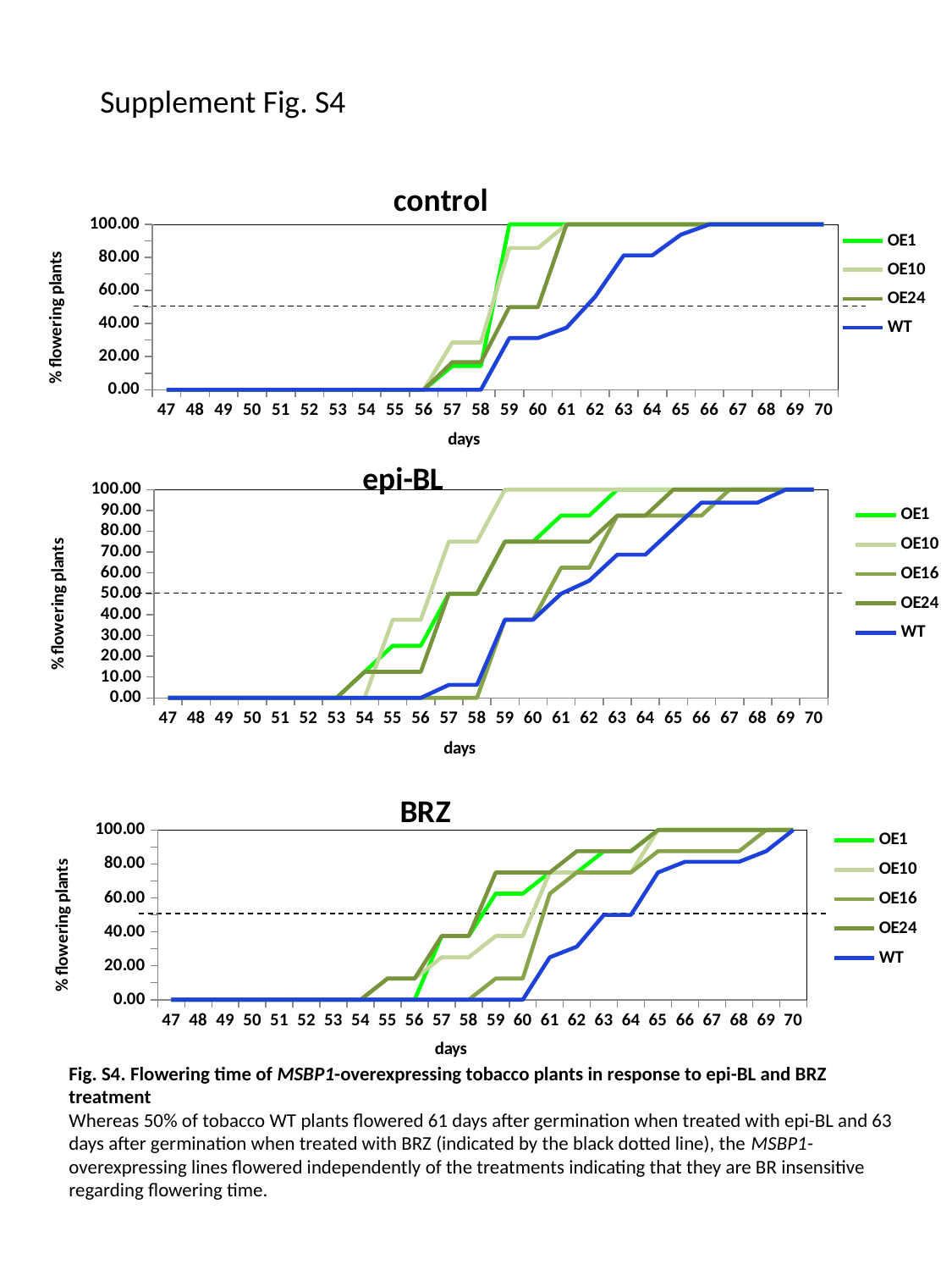

Supplement Fig. S4
### Chart: control
| Category | OE1 | OE10 | OE24 | WT |
|---|---|---|---|---|
| 47 | 0.0 | 0.0 | 0.0 | 0.0 |
| 48 | 0.0 | 0.0 | 0.0 | 0.0 |
| 49 | 0.0 | 0.0 | 0.0 | 0.0 |
| 50 | 0.0 | 0.0 | 0.0 | 0.0 |
| 51 | 0.0 | 0.0 | 0.0 | 0.0 |
| 52 | 0.0 | 0.0 | 0.0 | 0.0 |
| 53 | 0.0 | 0.0 | 0.0 | 0.0 |
| 54 | 0.0 | 0.0 | 0.0 | 0.0 |
| 55 | 0.0 | 0.0 | 0.0 | 0.0 |
| 56 | 0.0 | 0.0 | 0.0 | 0.0 |
| 57 | 14.285714285714292 | 28.57142857142857 | 16.66666666666666 | 0.0 |
| 58 | 14.285714285714292 | 28.57142857142857 | 16.66666666666666 | 0.0 |
| 59 | 100.0 | 85.71428571428572 | 50.0 | 31.25 |
| 60 | 100.0 | 85.71428571428572 | 50.0 | 31.25 |
| 61 | 100.0 | 100.0 | 100.0 | 37.5 |
| 62 | 100.0 | 100.0 | 100.0 | 56.25 |
| 63 | 100.0 | 100.0 | 100.0 | 81.25 |
| 64 | 100.0 | 100.0 | 100.0 | 81.25 |
| 65 | 100.0 | 100.0 | 100.0 | 93.75 |
| 66 | 100.0 | 100.0 | 100.0 | 100.0 |
| 67 | 100.0 | 100.0 | 100.0 | 100.0 |
| 68 | 100.0 | 100.0 | 100.0 | 100.0 |
| 69 | 100.0 | 100.0 | 100.0 | 100.0 |
| 70 | 100.0 | 100.0 | 100.0 | 100.0 |
### Chart: epi-BL
| Category | OE1 | OE10 | OE16 | OE24 | WT |
|---|---|---|---|---|---|
| 47 | 0.0 | 0.0 | 0.0 | 0.0 | 0.0 |
| 48 | 0.0 | 0.0 | 0.0 | 0.0 | 0.0 |
| 49 | 0.0 | 0.0 | 0.0 | 0.0 | 0.0 |
| 50 | 0.0 | 0.0 | 0.0 | 0.0 | 0.0 |
| 51 | 0.0 | 0.0 | 0.0 | 0.0 | 0.0 |
| 52 | 0.0 | 0.0 | 0.0 | 0.0 | 0.0 |
| 53 | 0.0 | 0.0 | 0.0 | 0.0 | 0.0 |
| 54 | 12.5 | 0.0 | 0.0 | 12.5 | 0.0 |
| 55 | 25.0 | 37.5 | 0.0 | 12.5 | 0.0 |
| 56 | 25.0 | 37.5 | 0.0 | 12.5 | 0.0 |
| 57 | 50.0 | 75.0 | 0.0 | 50.0 | 6.25 |
| 58 | 50.0 | 75.0 | 0.0 | 50.0 | 6.25 |
| 59 | 75.0 | 100.0 | 37.5 | 75.0 | 37.5 |
| 60 | 75.0 | 100.0 | 37.5 | 75.0 | 37.5 |
| 61 | 87.5 | 100.0 | 62.5 | 75.0 | 50.0 |
| 62 | 87.5 | 100.0 | 62.5 | 75.0 | 56.25 |
| 63 | 100.0 | 100.0 | 87.5 | 87.5 | 68.75 |
| 64 | 100.0 | 100.0 | 87.5 | 87.5 | 68.75 |
| 65 | 100.0 | 100.0 | 87.5 | 100.0 | 81.25 |
| 66 | 100.0 | 100.0 | 87.5 | 100.0 | 93.75 |
| 67 | 100.0 | 100.0 | 100.0 | 100.0 | 93.75 |
| 68 | 100.0 | 100.0 | 100.0 | 100.0 | 93.75 |
| 69 | 100.0 | 100.0 | 100.0 | 100.0 | 100.0 |
| 70 | 100.0 | 100.0 | 100.0 | 100.0 | 100.0 |
### Chart: BRZ
| Category | OE1 | OE10 | OE16 | OE24 | WT |
|---|---|---|---|---|---|
| 47 | 0.0 | 0.0 | 0.0 | 0.0 | 0.0 |
| 48 | 0.0 | 0.0 | 0.0 | 0.0 | 0.0 |
| 49 | 0.0 | 0.0 | 0.0 | 0.0 | 0.0 |
| 50 | 0.0 | 0.0 | 0.0 | 0.0 | 0.0 |
| 51 | 0.0 | 0.0 | 0.0 | 0.0 | 0.0 |
| 52 | 0.0 | 0.0 | 0.0 | 0.0 | 0.0 |
| 53 | 0.0 | 0.0 | 0.0 | 0.0 | 0.0 |
| 54 | 0.0 | 0.0 | 0.0 | 0.0 | 0.0 |
| 55 | 0.0 | 12.5 | 0.0 | 12.5 | 0.0 |
| 56 | 0.0 | 12.5 | 0.0 | 12.5 | 0.0 |
| 57 | 37.5 | 25.0 | 0.0 | 37.5 | 0.0 |
| 58 | 37.5 | 25.0 | 0.0 | 37.5 | 0.0 |
| 59 | 62.5 | 37.5 | 12.5 | 75.0 | 0.0 |
| 60 | 62.5 | 37.5 | 12.5 | 75.0 | 0.0 |
| 61 | 75.0 | 75.0 | 62.5 | 75.0 | 25.0 |
| 62 | 75.0 | 75.0 | 75.0 | 87.5 | 31.25 |
| 63 | 87.5 | 75.0 | 75.0 | 87.5 | 50.0 |
| 64 | 87.5 | 75.0 | 75.0 | 87.5 | 50.0 |
| 65 | 100.0 | 100.0 | 87.5 | 100.0 | 75.0 |
| 66 | 100.0 | 100.0 | 87.5 | 100.0 | 81.25 |
| 67 | 100.0 | 100.0 | 87.5 | 100.0 | 81.25 |
| 68 | 100.0 | 100.0 | 87.5 | 100.0 | 81.25 |
| 69 | 100.0 | 100.0 | 100.0 | 100.0 | 87.5 |
| 70 | 100.0 | 100.0 | 100.0 | 100.0 | 100.0 |Fig. S4. Flowering time of MSBP1-overexpressing tobacco plants in response to epi-BL and BRZ treatment
Whereas 50% of tobacco WT plants flowered 61 days after germination when treated with epi-BL and 63 days after germination when treated with BRZ (indicated by the black dotted line), the MSBP1-overexpressing lines flowered independently of the treatments indicating that they are BR insensitive regarding flowering time.

## Slide 5
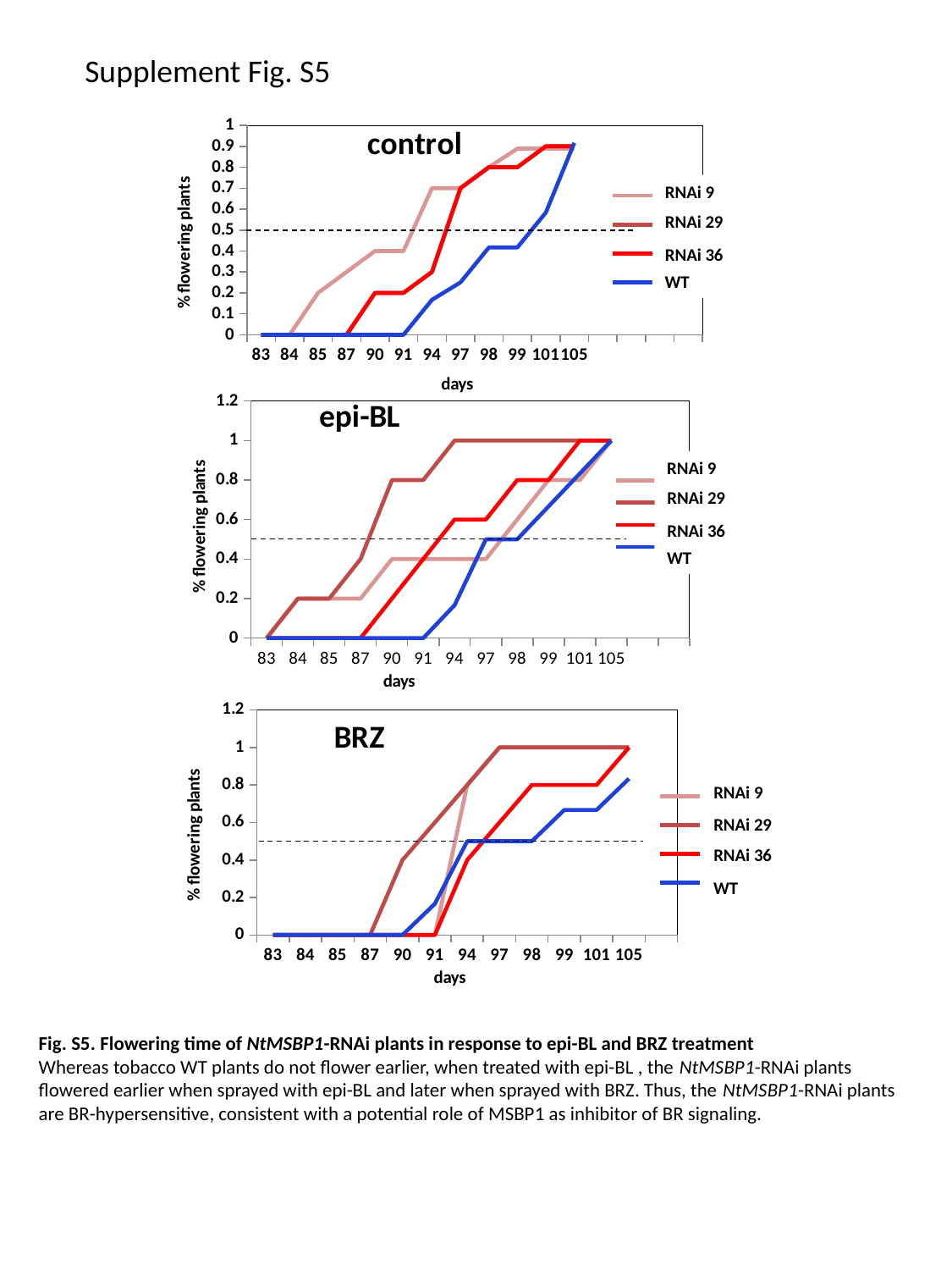

Supplement Fig. S5
[unsupported chart]
RNAi 9
RNAi 29
RNAi 36
WT
### Chart: epi-BL
| Category | | | | |
|---|---|---|---|---|
| 83 | 0.0 | 0.0 | 0.0 | 0.0 |
| 84 | 0.2 | 0.2 | 0.0 | 0.0 |
| 85 | 0.2 | 0.2 | 0.0 | 0.0 |
| 87 | 0.2 | 0.4 | 0.0 | 0.0 |
| 90 | 0.4 | 0.8 | 0.2 | 0.0 |
| 91 | 0.4 | 0.8 | 0.4 | 0.0 |
| 94 | 0.4 | 1.0 | 0.6000000000000011 | 0.16666666666666696 |
| 97 | 0.4 | 1.0 | 0.6000000000000011 | 0.5 |
| 98 | 0.6000000000000011 | 1.0 | 0.8 | 0.5 |
| 99 | 0.8 | 1.0 | 0.8 | 0.6666666666666671 |
| 101 | 0.8 | 1.0 | 1.0 | 0.833333333333334 |
| 105 | 1.0 | 1.0 | 1.0 | 1.0 |RNAi 9
RNAi 29
RNAi 36
WT
### Chart: BRZ
| Category | | | | |
|---|---|---|---|---|
| 83 | 0.0 | 0.0 | 0.0 | 0.0 |
| 84 | 0.0 | 0.0 | 0.0 | 0.0 |
| 85 | 0.0 | 0.0 | 0.0 | 0.0 |
| 87 | 0.0 | 0.0 | 0.0 | 0.0 |
| 90 | 0.0 | 0.4 | 0.0 | 0.0 |
| 91 | 0.0 | 0.6000000000000011 | 0.0 | 0.16666666666666696 |
| 94 | 0.8 | 0.8 | 0.4 | 0.5 |
| 97 | 1.0 | 1.0 | 0.6000000000000011 | 0.5 |
| 98 | 1.0 | 1.0 | 0.8 | 0.5 |
| 99 | 1.0 | 1.0 | 0.8 | 0.6666666666666671 |
| 101 | 1.0 | 1.0 | 0.8 | 0.6666666666666671 |
| 105 | 1.0 | 1.0 | 1.0 | 0.833333333333334 |
| | None | None | None | None |RNAi 9
RNAi 29
RNAi 36
WT
Fig. S5. Flowering time of NtMSBP1-RNAi plants in response to epi-BL and BRZ treatment
Whereas tobacco WT plants do not flower earlier, when treated with epi-BL , the NtMSBP1-RNAi plants flowered earlier when sprayed with epi-BL and later when sprayed with BRZ. Thus, the NtMSBP1-RNAi plants are BR-hypersensitive, consistent with a potential role of MSBP1 as inhibitor of BR signaling.

## Slide 6
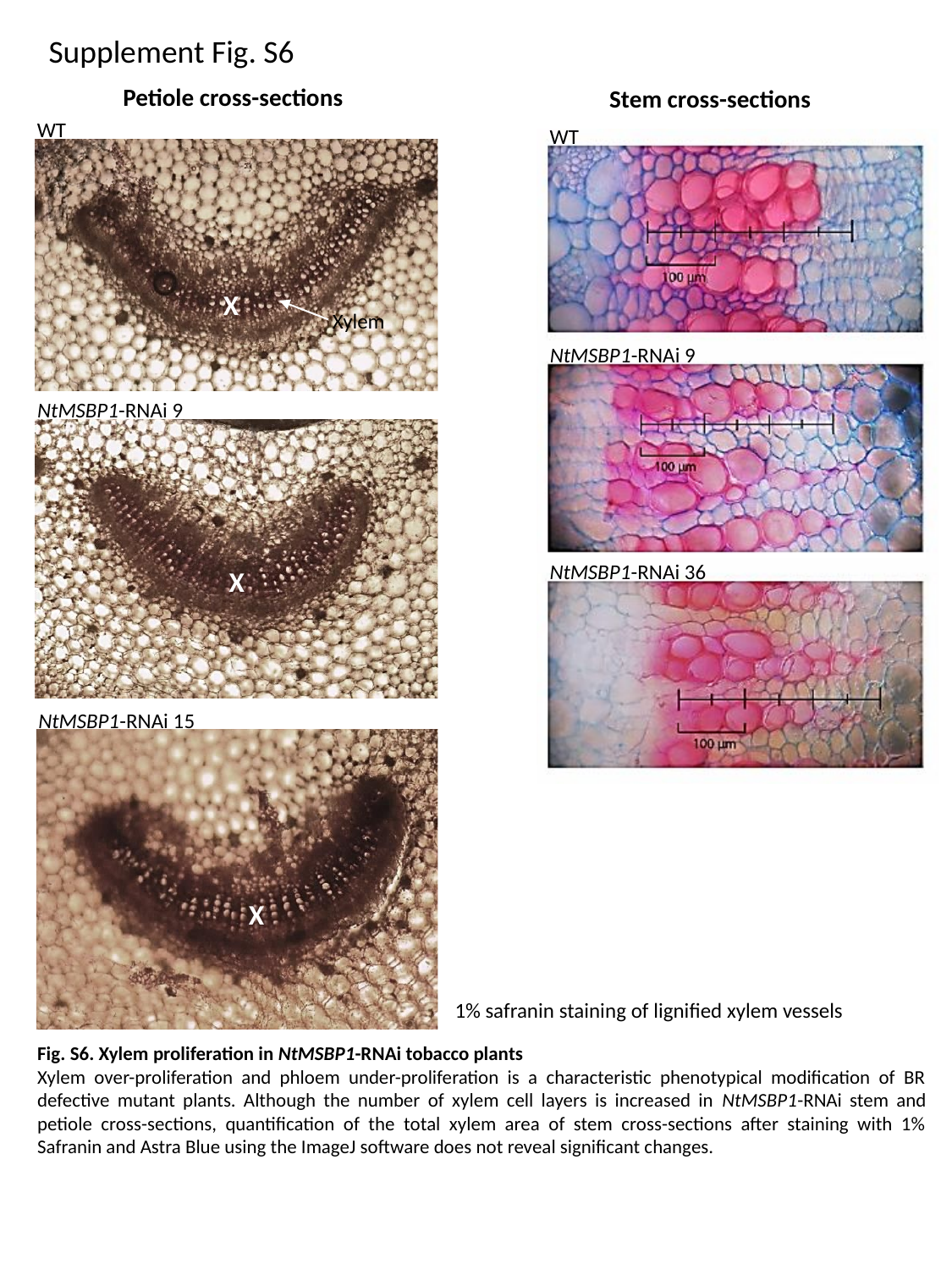

Supplement Fig. S6
Petiole cross-sections
Stem cross-sections
WT
WT
X
Xylem
NtMSBP1-RNAi 9
NtMSBP1-RNAi 9
NtMSBP1-RNAi 36
X
NtMSBP1-RNAi 15
X
1% safranin staining of lignified xylem vessels
Fig. S6. Xylem proliferation in NtMSBP1-RNAi tobacco plants
Xylem over-proliferation and phloem under-proliferation is a characteristic phenotypical modification of BR defective mutant plants. Although the number of xylem cell layers is increased in NtMSBP1-RNAi stem and petiole cross-sections, quantification of the total xylem area of stem cross-sections after staining with 1% Safranin and Astra Blue using the ImageJ software does not reveal significant changes.

## Slide 7
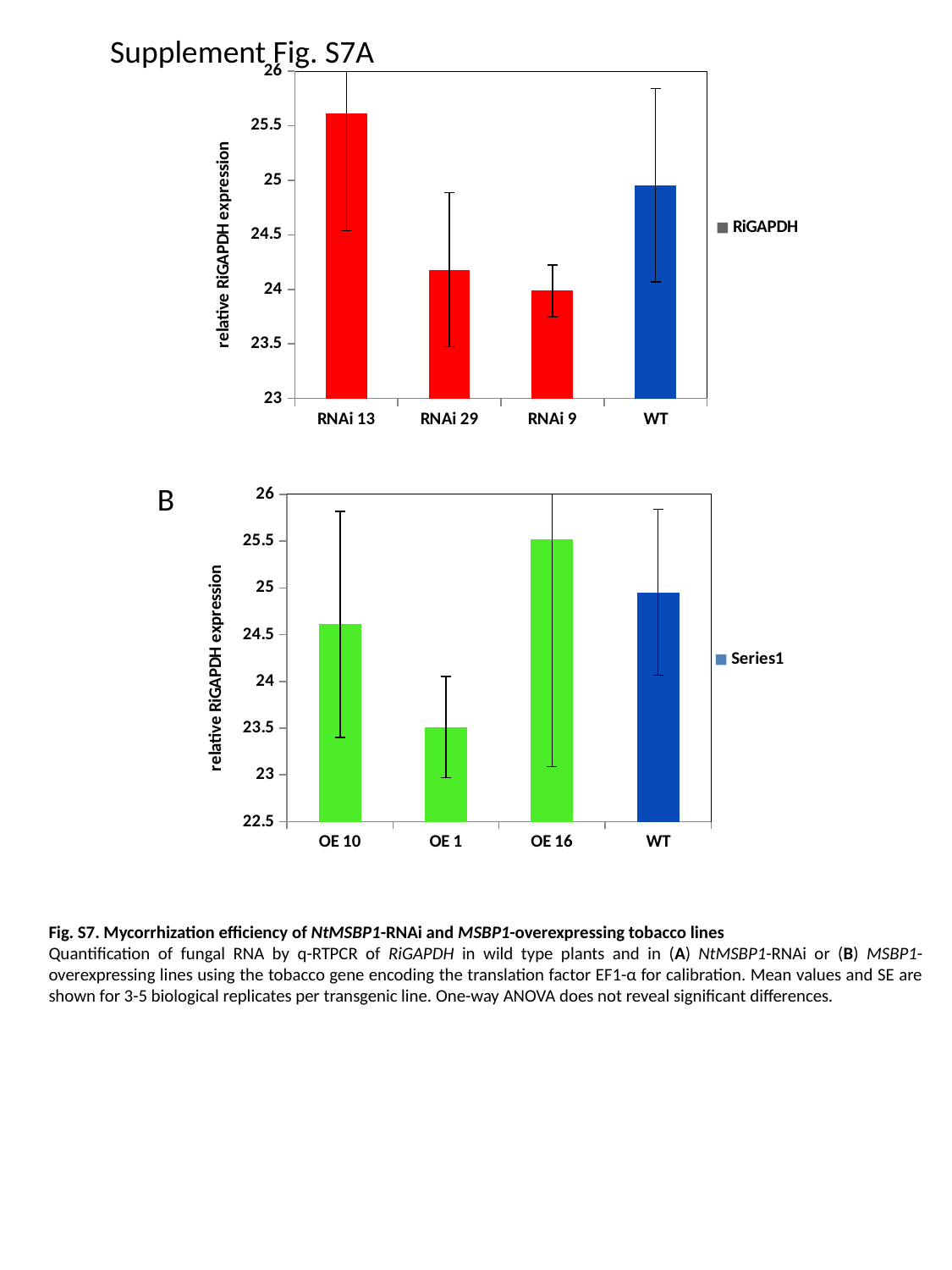

Supplement Fig. S7A
### Chart
| Category | |
|---|---|
| RNAi 13 | 25.613000000000003 |
| RNAi 29 | 24.18 |
| RNAi 9 | 23.986999999999984 |
| WT | 24.952999999999996 |B
### Chart
| Category | |
|---|---|
| OE 10 | 24.610000000000003 |
| OE 1 | 23.51 |
| OE 16 | 25.518 |
| WT | 24.952999999999996 |Fig. S7. Mycorrhization efficiency of NtMSBP1-RNAi and MSBP1-overexpressing tobacco lines
Quantification of fungal RNA by q-RTPCR of RiGAPDH in wild type plants and in (A) NtMSBP1-RNAi or (B) MSBP1-overexpressing lines using the tobacco gene encoding the translation factor EF1-α for calibration. Mean values and SE are shown for 3-5 biological replicates per transgenic line. One-way ANOVA does not reveal significant differences.

## Slide 8
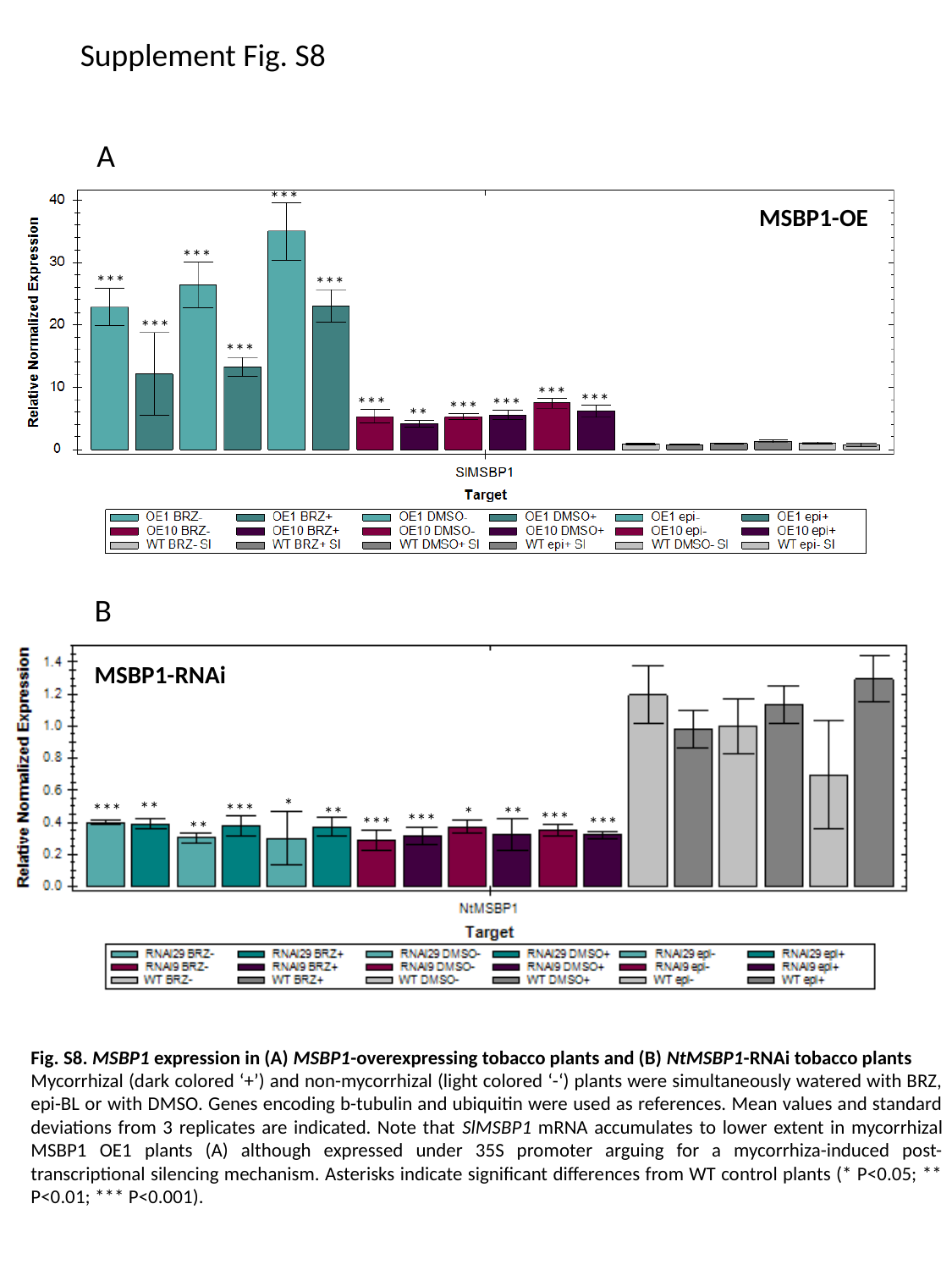

Supplement Fig. S8
A
***
MSBP1-OE
***
***
***
***
***
***
***
***
***
***
**
B
MSBP1-RNAi
*
**
***
***
**
**
*
***
***
***
***
**
Fig. S8. MSBP1 expression in (A) MSBP1-overexpressing tobacco plants and (B) NtMSBP1-RNAi tobacco plants
Mycorrhizal (dark colored ‘+’) and non-mycorrhizal (light colored ‘-‘) plants were simultaneously watered with BRZ, epi-BL or with DMSO. Genes encoding b-tubulin and ubiquitin were used as references. Mean values and standard deviations from 3 replicates are indicated. Note that SlMSBP1 mRNA accumulates to lower extent in mycorrhizal MSBP1 OE1 plants (A) although expressed under 35S promoter arguing for a mycorrhiza-induced post-transcriptional silencing mechanism. Asterisks indicate significant differences from WT control plants (* P<0.05; ** P<0.01; *** P<0.001).

## Slide 9
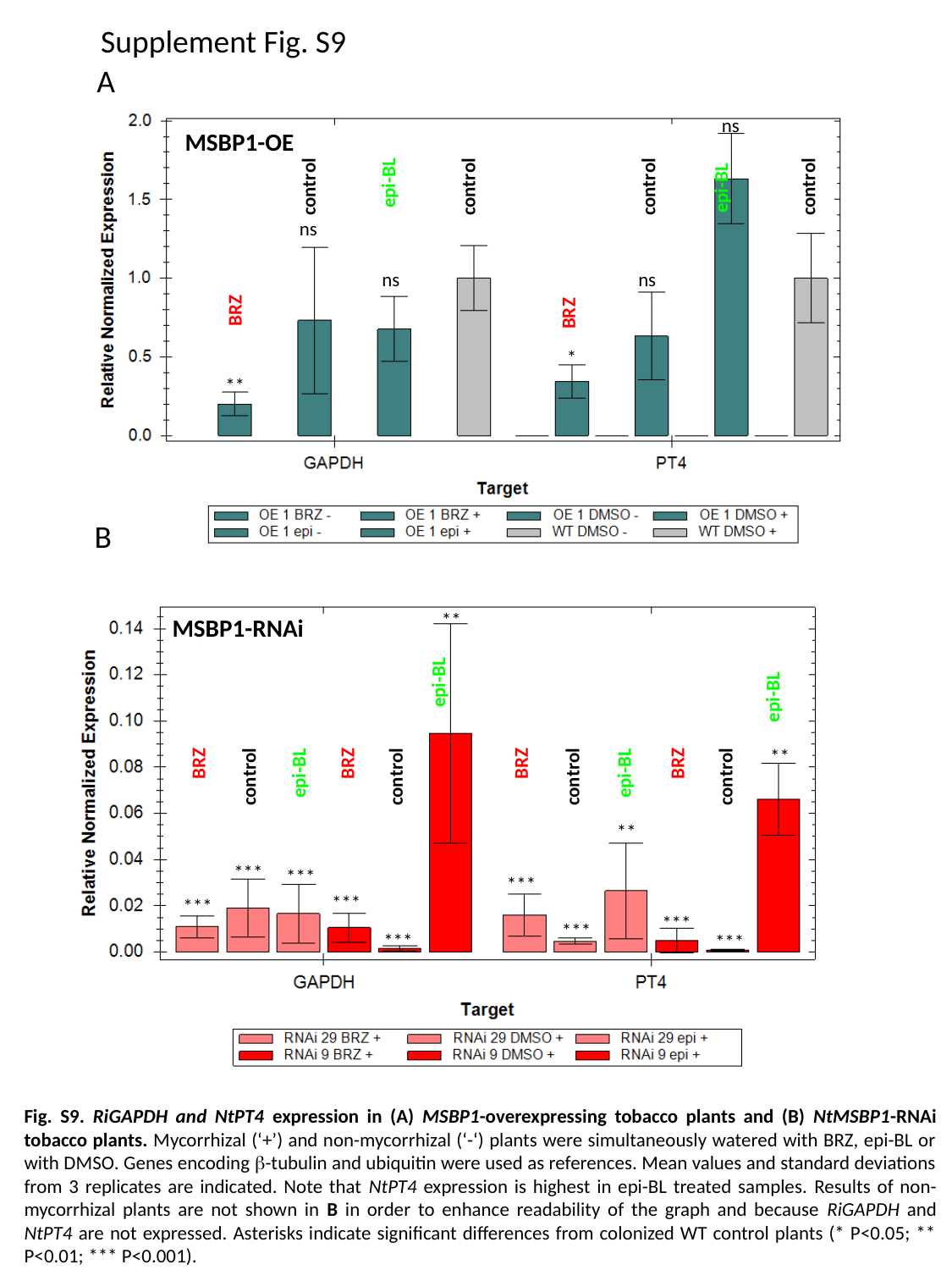

Supplement Fig. S9
A
ns
MSBP1-OE
epi-BL
control
control
control
control
epi-BL
ns
ns
ns
BRZ
BRZ
*
**
B
**
MSBP1-RNAi
epi-BL
epi-BL
**
BRZ
BRZ
BRZ
BRZ
epi-BL
epi-BL
control
control
control
control
**
***
***
***
***
***
***
***
***
***
Fig. S9. RiGAPDH and NtPT4 expression in (A) MSBP1-overexpressing tobacco plants and (B) NtMSBP1-RNAi tobacco plants. Mycorrhizal (‘+’) and non-mycorrhizal (‘-‘) plants were simultaneously watered with BRZ, epi-BL or with DMSO. Genes encoding b-tubulin and ubiquitin were used as references. Mean values and standard deviations from 3 replicates are indicated. Note that NtPT4 expression is highest in epi-BL treated samples. Results of non-mycorrhizal plants are not shown in B in order to enhance readability of the graph and because RiGAPDH and NtPT4 are not expressed. Asterisks indicate significant differences from colonized WT control plants (* P<0.05; ** P<0.01; *** P<0.001).

## Slide 10
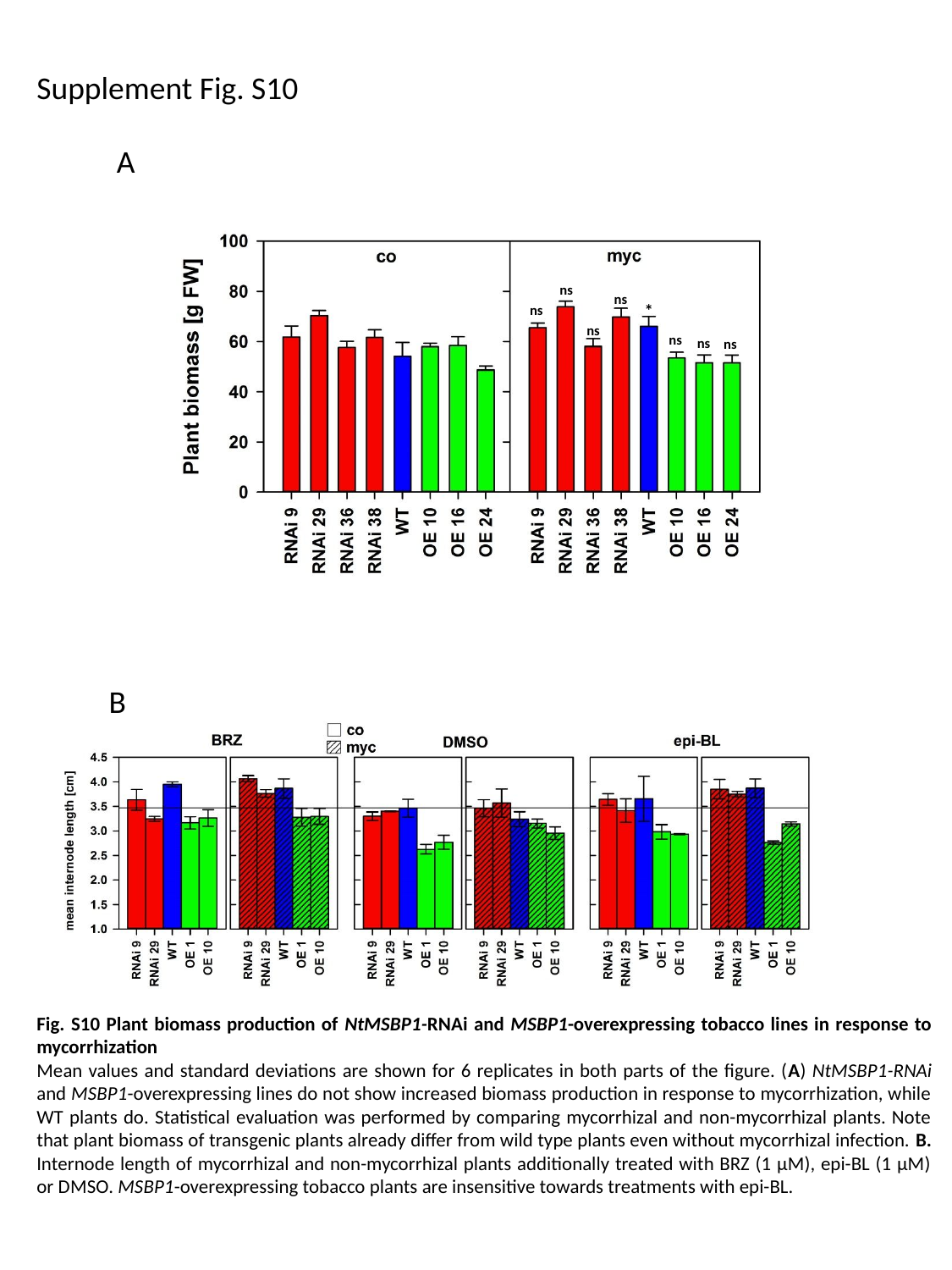

Supplement Fig. S10
A
ns
ns
*
ns
ns
ns
ns
ns
B
Fig. S10 Plant biomass production of NtMSBP1-RNAi and MSBP1-overexpressing tobacco lines in response to mycorrhization
Mean values and standard deviations are shown for 6 replicates in both parts of the figure. (A) NtMSBP1-RNAi and MSBP1-overexpressing lines do not show increased biomass production in response to mycorrhization, while WT plants do. Statistical evaluation was performed by comparing mycorrhizal and non-mycorrhizal plants. Note that plant biomass of transgenic plants already differ from wild type plants even without mycorrhizal infection. B. Internode length of mycorrhizal and non-mycorrhizal plants additionally treated with BRZ (1 µM), epi-BL (1 µM) or DMSO. MSBP1-overexpressing tobacco plants are insensitive towards treatments with epi-BL.

## Slide 11
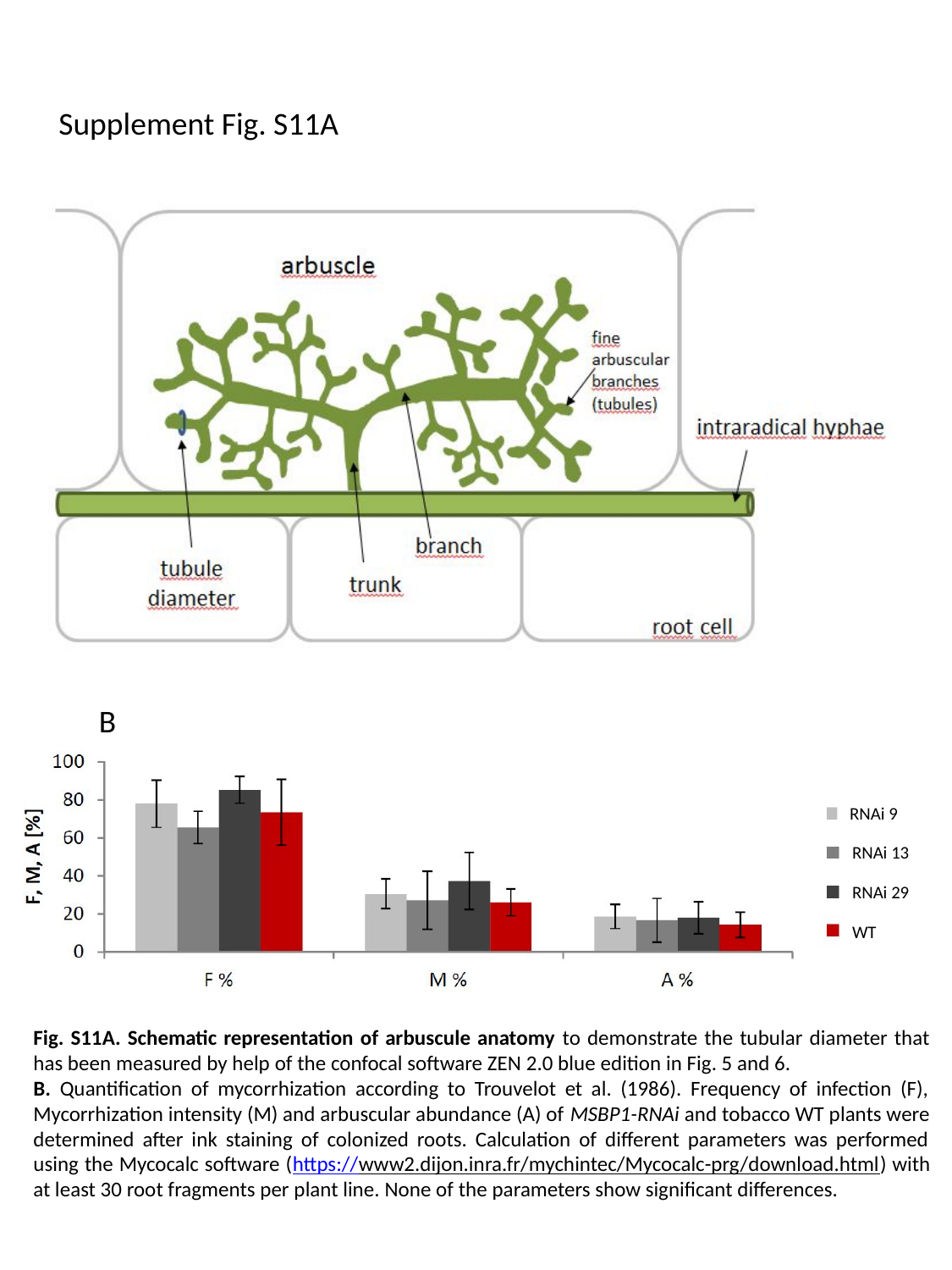

Supplement Fig. S11A
B
RNAi 9
RNAi 13
RNAi 29
WT
Fig. S11A. Schematic representation of arbuscule anatomy to demonstrate the tubular diameter that has been measured by help of the confocal software ZEN 2.0 blue edition in Fig. 5 and 6.
B. Quantification of mycorrhization according to Trouvelot et al. (1986). Frequency of infection (F), Mycorrhization intensity (M) and arbuscular abundance (A) of MSBP1-RNAi and tobacco WT plants were determined after ink staining of colonized roots. Calculation of different parameters was performed using the Mycocalc software (https://www2.dijon.inra.fr/mychintec/Mycocalc-prg/download.html) with at least 30 root fragments per plant line. None of the parameters show significant differences.

## Slide 12
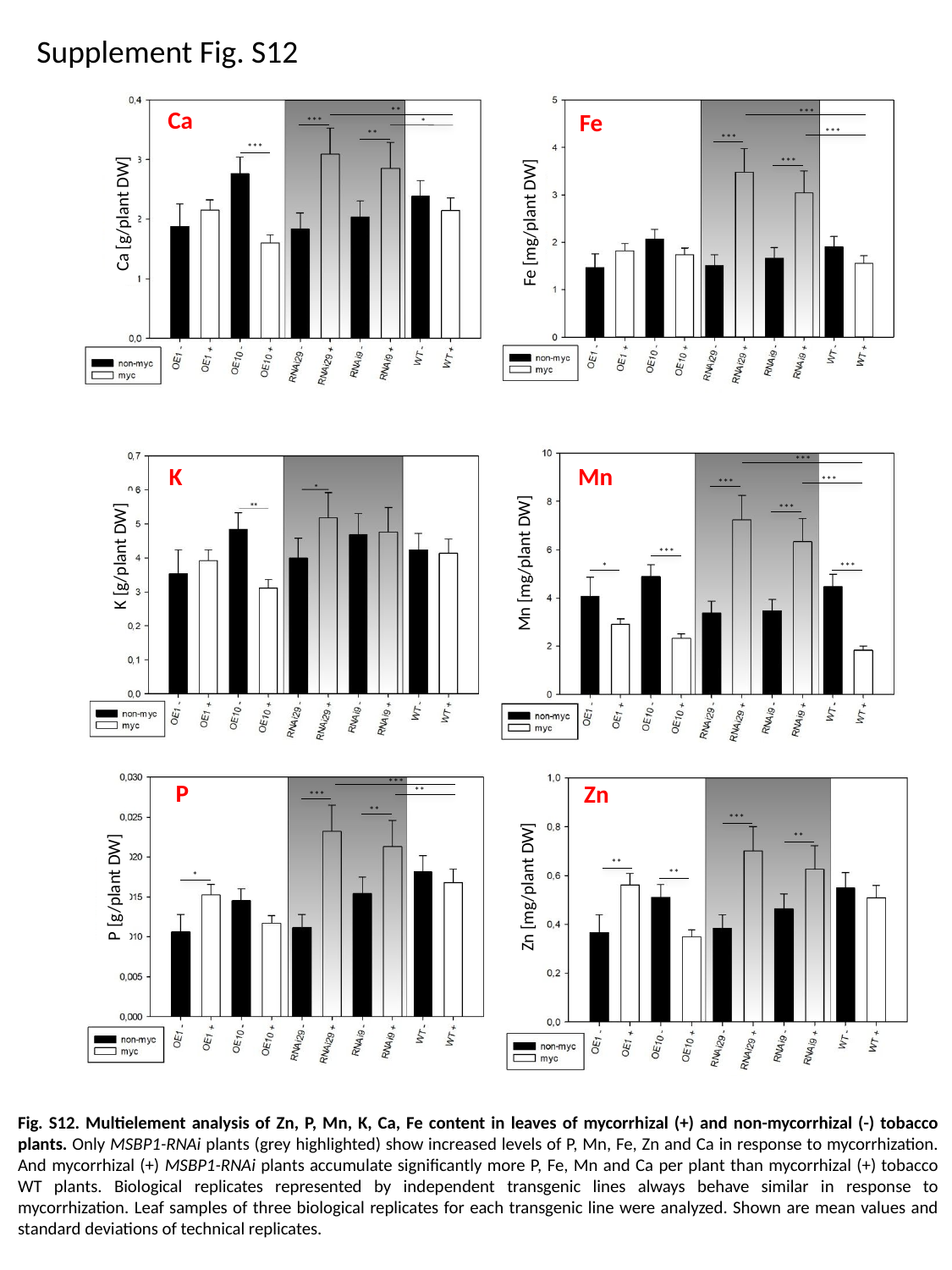

Supplement Fig. S12
Ca
**
***
Fe
***
*
***
**
***
***
***
Ca [g/plant DW]
Fe [mg/plant DW]
***
K
Mn
***
***
***
K [g/plant DW]
***
Mn [mg/plant DW]
***
*
***
P
Zn
**
***
**
***
**
**
**
*
Zn [mg/plant DW]
P [g/plant DW]
Fig. S12. Multielement analysis of Zn, P, Mn, K, Ca, Fe content in leaves of mycorrhizal (+) and non-mycorrhizal (-) tobacco plants. Only MSBP1-RNAi plants (grey highlighted) show increased levels of P, Mn, Fe, Zn and Ca in response to mycorrhization. And mycorrhizal (+) MSBP1-RNAi plants accumulate significantly more P, Fe, Mn and Ca per plant than mycorrhizal (+) tobacco WT plants. Biological replicates represented by independent transgenic lines always behave similar in response to mycorrhization. Leaf samples of three biological replicates for each transgenic line were analyzed. Shown are mean values and standard deviations of technical replicates.

## Slide 13
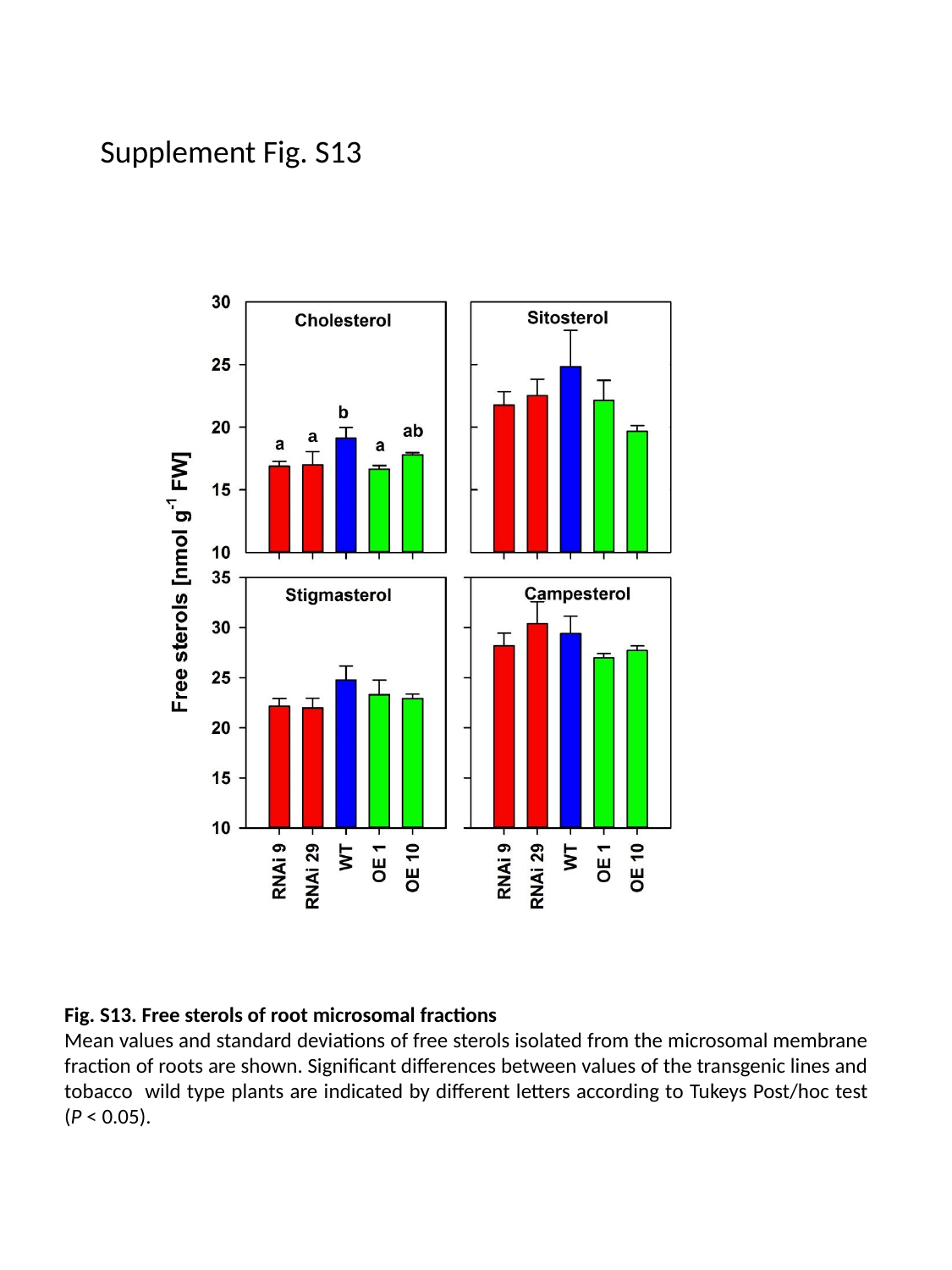

Supplement Fig. S13
a
Fig. S13. Free sterols of root microsomal fractions
Mean values and standard deviations of free sterols isolated from the microsomal membrane fraction of roots are shown. Significant differences between values of the transgenic lines and tobacco wild type plants are indicated by different letters according to Tukeys Post/hoc test (P < 0.05).

## Slide 14
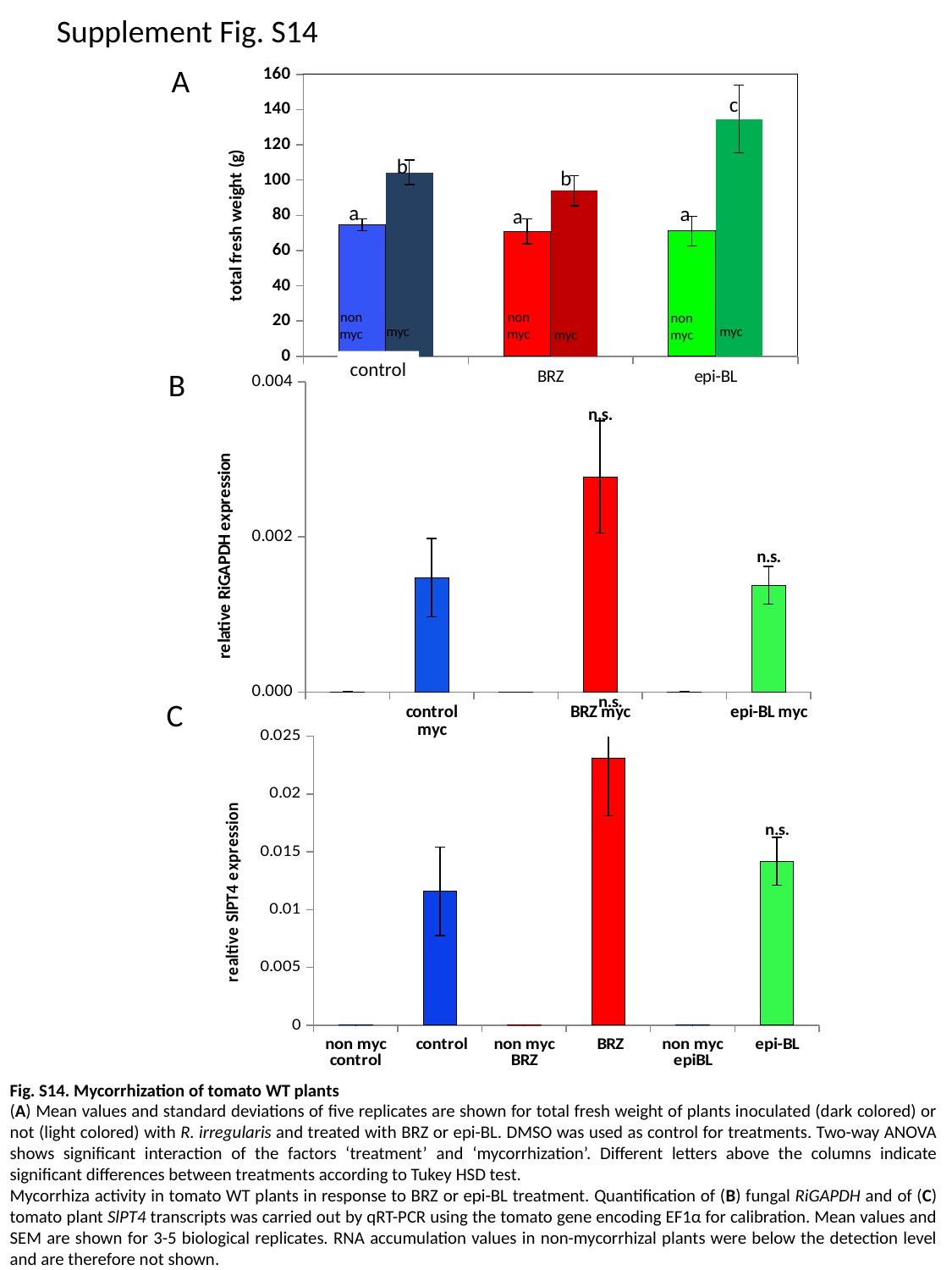

Supplement Fig. S14
A
### Chart
| Category | | |
|---|---|---|
| DMSO | 74.6 | 104.4 |
| BRZ | 70.8 | 94.0 |
| epi-BL | 71.0 | 134.6 |c
b
b
a
a
a
non
myc
non
myc
non
myc
myc
myc
myc
control
B
### Chart
| Category | |
|---|---|
| | 1.67881e-06 |
| control myc | 0.001476816 |
| | 9.79001e-09 |
| BRZ myc | 0.002772756 |
| | 2.15288e-06 |
| epi-BL myc | 0.001376189 |
### Chart
| Category |
|---|C
### Chart
| Category | |
|---|---|
| non myc control | 3.574343448840153e-06 |
| control | 0.0115909375581341 |
| non myc BRZ | 1.9022050776374538e-06 |
| BRZ | 0.023088117279486325 |
| non myc epiBL | 7.807109447417903e-06 |
| epi-BL | 0.014193997510718329 |
Fig. S14. Mycorrhization of tomato WT plants
(A) Mean values and standard deviations of five replicates are shown for total fresh weight of plants inoculated (dark colored) or not (light colored) with R. irregularis and treated with BRZ or epi-BL. DMSO was used as control for treatments. Two-way ANOVA shows significant interaction of the factors ‘treatment’ and ‘mycorrhization’. Different letters above the columns indicate significant differences between treatments according to Tukey HSD test.
Mycorrhiza activity in tomato WT plants in response to BRZ or epi-BL treatment. Quantification of (B) fungal RiGAPDH and of (C) tomato plant SlPT4 transcripts was carried out by qRT-PCR using the tomato gene encoding EF1α for calibration. Mean values and SEM are shown for 3-5 biological replicates. RNA accumulation values in non-mycorrhizal plants were below the detection level and are therefore not shown.

## Slide 15
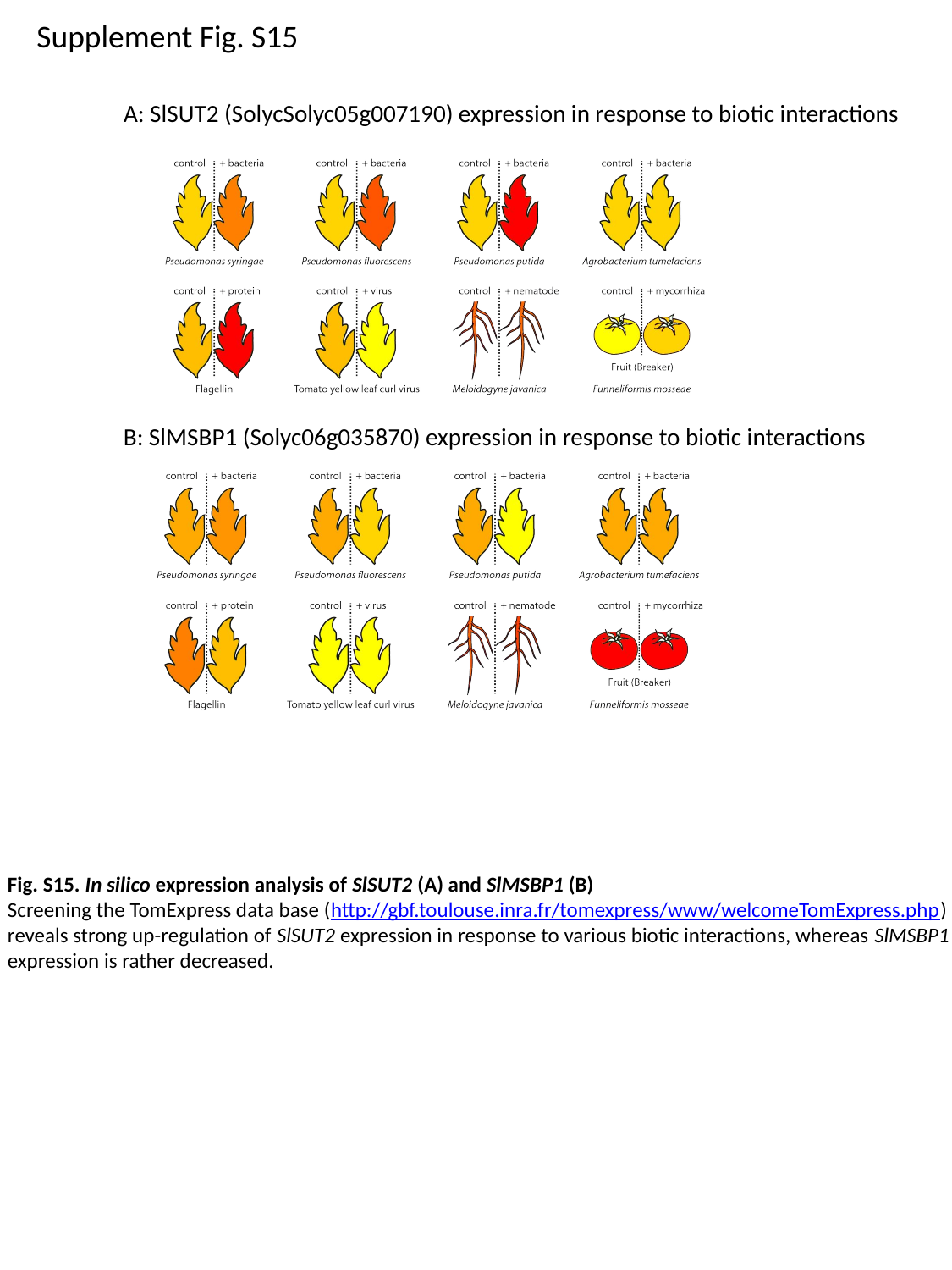

Supplement Fig. S15
A: SlSUT2 (SolycSolyc05g007190) expression in response to biotic interactions
B: SlMSBP1 (Solyc06g035870) expression in response to biotic interactions
Fig. S15. In silico expression analysis of SlSUT2 (A) and SlMSBP1 (B)
Screening the TomExpress data base (http://gbf.toulouse.inra.fr/tomexpress/www/welcomeTomExpress.php) reveals strong up-regulation of SlSUT2 expression in response to various biotic interactions, whereas SlMSBP1 expression is rather decreased.
